# Lifestyle Interventions and Pharmacological Adherence in the Management of Hypertension Among Community‐Dwelling Older Adults: A Systematic Review

**DOI:** 10.1155/nrp/7044570

**Published:** 2026-05-28

**Authors:** Khanisorn Ransinyo, Samoraphop Banharak, Chakkarin Sommana

**Affiliations:** ^1^ Department of Gerontological Nursing, Faculty of Nursing, Khon Kaen University, Khon Kaen, Thailand, kku.ac.th

**Keywords:** aged, aging in place, disease management, hypertension, medication adherence

## Abstract

Hypertension prevalence is rising, particularly among older adults, who are often challenging to manage due to various limitations. Uncontrolled hypertension contributes significantly to complications and disease burdens, including myocardial ischemia and stroke, ultimately impairing functional ability and diminishing overall quality of life. This study aimed to review the components, details, and effects of hypertension management interventions among older adults. The protocol was prospectively registered in PROSPERO for verification and guidance. We searched across eleven databases: PubMed, CINAHL, Scopus, ProQuest, PsycINFO, AgeLine, ScienceDirect, ThaiList, ThaiJO, Cochrane Library, and OneSearch. Studies among older adults published between January 2019 and February 2025 were included in this review. The initial results included 15,024 identified records; 16 studies met the inclusion criteria, comprising 10 randomized controlled trials and six quasi‐experimental studies, with a total of 1857 participants. The multicomponent interventions produced the largest and most consistent blood pressure reductions, with SBP decreases up to −21.90 mmHg and DBP up to −9.46 mmHg. These effects were associated with moderate‐duration programs (10–16 weeks), repeated structured contact, and reinforcement mechanisms. In contrast, lower‐intensity or single‐component interventions yielded smaller or inconsistent effects. Therefore, the findings indicate a clear gradient of effectiveness, with greater SBP reduction associated with increasing intervention complexity and integration. These results highlight the importance of designing interventions that move beyond single‐component education toward multilevel behavioral systems, particularly for older adults with diverse capabilities and support needs. The secondary outcomes demonstrated a similar but less pronounced gradient compared to blood pressure, with multicomponent interventions consistently producing the most favorable improvements in medication adherence, health behaviors, and hypertension‐related knowledge. In conclusion, multicomponent interventions with high‐intensity interventions produce the most consistent improvements in blood pressure and related behaviors among older adults, highlighting that effectiveness is driven by integrated strategies, reinforcement, and implementation quality rather than single‐component approaches.

## 1. Introduction

Hypertension remains one of the most prevalent chronic conditions among older adults and a major contributor to cardiovascular morbidity and mortality worldwide [1]. Age‐related vascular changes [2], which include arterial stiffness and endothelial dysfunction, increase susceptibility to systolic hypertension and related complications such as stroke, myocardial infarction, heart failure, and chronic kidney disease [3–5]. Although blood pressure control substantially reduces cardiovascular risk, older adults present unique clinical challenges due to multimorbidity, frailty, polypharmacy, and age‐related functional decline [6, 7]. A comprehensive understanding of this increased prevalence and its potential deleterious consequences in this population is essential for developing effective management and prevention strategies [3, 8, 9].

Global population aging has intensified the burden of hypertension, particularly among adults aged 65 years and older [10, 11]. Despite advances in pharmacotherapy, blood pressure control rates remain suboptimal [12], especially in low‐ and middle‐income countries [7, 11, 13]. These gaps underscore the need for effective, scalable, and age‐appropriate management strategies that extend beyond pharmacological treatment alone, especially within aging‐in‐place frameworks [11, 14, 15].

Hypertension management in older adults involves both pharmacological and nonpharmacological approaches [15, 16]. However, pharmacological management in this population is complicated by increased vulnerability to adverse effects, complex regimens, and adherence challenges related to cognitive impairment and polypharmacy [16]. Nonpharmacological components can enhance blood pressure control by targeting modifiable behavioral and physiological pathways that pharmacotherapy alone may not fully address [17]. Dietary modification and sodium reduction improve vascular function and reduce volume load; regular physical activity enhances endothelial function and arterial compliance; stress management may attenuate sympathetic activation; and structured medication adherence support improves treatment consistency [18, 19]. In addition, patient education and self‐monitoring promote self‐efficacy, appropriate medication use, and timely adjustment of care. Together, these components may synergistically improve blood pressure control while potentially reducing medication burden and minimizing treatment‐related adverse effects in community‐dwelling older adults [3].

Numerous interventions targeting hypertension have been evaluated in the general adult population, and several systematic reviews have synthesized digital, text messaging, and lifestyle‐based interventions [20, 21]. However, most prior reviews have either (1) included broad adult age ranges without isolating older adults, (2) focused narrowly on single delivery modalities (e.g., mobile health or text messaging), or (3) primarily evaluated overall effectiveness without analyzing how intervention components were combined and delivered. Consequently, existing evidence provides limited insight into which intervention components and details are effective for promoting hypertension management among community‐dwelling older adults. In particular, it remains unclear which program components and details—such as nutrition, exercise, stress management, medication adherence support, and self‐monitoring—should be incorporated, as well as which delivery strategies, including education, consultation, individual face‐to‐face intervention, self‐monitoring, and social support, are effective in improving blood pressure outcomes in this population. This gap limits the ability to determine which intervention component combinations are most appropriate and scalable for older populations with age‐related physiological and functional complexities.

In conclusion, hypertension in older adults is an essential contributor to significant cardiovascular and cognitive risks. Effective management requires not only blood pressure control but also careful consideration of medication‐related adverse effects [16]. However, no prior systematic review has systematically examined how pharmacological and nonpharmacological components are structured and delivered together within hypertension programs specifically designed for community‐dwelling older adults. Existing reviews have either synthesized broad adult populations, focused on single‐intervention modalities, or evaluated overall effectiveness without analyzing the underlying configuration of components and delivery strategies [22]. As a result, it remains unclear which combinations of lifestyle modification, medication adherence support, self‐monitoring, and behavioral strategies, when delivered through education, consultation, monitoring, or social support that are most suitable and effective for improving blood pressure in older adults. Therefore, this systematic review primarily aimed to identify the intervention components and delivery strategies used in these programs and to compare the intervention groups reporting effects on medication adherence, health behaviors, hypertension‐related knowledge, psychological outcomes, and selected cardiovascular biomarkers.

## 2. Aims

The primary aim was to identify and categorize the components and delivery strategies of nonpharmacological interventions on blood pressure. The secondary aim was to compare the effects of these interventions on clinical and patient‐related outcomes among community‐dwelling older adults.

## 3. Methods

### 3.1. Design

We conducted this systematic review with a narrative detailing intervention outcome, following the PRISMA guidelines and using the Joanna Briggs Institute critical appraisal tools [23]. The protocol was prospectively registered with PROSPERO (No. CRD42024533008) to ensure methodological rigor and transparency.

### 3.2. Search Strategies

Keywords were identified for searching using the PICO framework: population, intervention, comparison, and outcomes. The population is community‐dwelling older adults who were diagnosed with hypertension. The intervention is a hypertension management intervention, the comparison is no intervention or usual care, and the outcomes are knowledge, health behaviors, beliefs, and biomarkers. The search terms for population included “aged,” “older adult,” “older person,” “older people,” “elderly,” “senior,” and “hypertension.” The search terms for interventions are “health education,” “health literacy,” “intervention,” “lifestyle,” “exercise,” “program,” “community‐based,” “telehealth,” and “chatbot.” The comparison search terms are “usual care,” “routine care,” “home visit,” and “routine suggestion.” Finally, the search terms for the primary outcomes were “blood pressure,” “systolic blood pressure,” and “diastolic blood pressure.” In addition, we also search for possible secondary outcomes that may be introduced by the hypertension management program, which consisted of “knowledge,” “health behaviors,” “beliefs,” “self‐care,” “self‐monitoring,” “self‐efficacy,” “coping,” “anxiety,” “awareness,” and “biomarkers.” The researchers used “OR” to connect wording within the concept. However, “AND” was used to connect words between concepts. The search statement was developed and published in PROSPERO to allow duplication and verification. The search used keywords and searched 11 databases: PubMed, CINAHL, SCOPUS, ProQuest, PsycINFO, AgeLine, ScienceDirect, ThaiList, ThaiJO, Cochrane Library, and OneSearch. We searched for studies published between January 2015 and February 2025. This 10‐year window was selected to align with the approximate cycle of major hypertension guideline updates and to capture the acceleration of contemporary delivery solutions (e.g., mobile health, telemonitoring, automated messaging) that have become increasingly embedded in community‐based hypertension management programs during the past decade. Finally, we planned to contact the principal researchers to request any missing data. However, we achieved all the necessary information without any contact.

### 3.3. Inclusion and Exclusion Criteria

The inclusion criteria comprised studies that (1) included participants in whom at least 80% were aged 60 years or older, ensuring that older adults represented the majority of the study population; (2) evaluated nonpharmacological interventions for hypertension management; (3) reported blood pressure outcomes or other clearly defined clinical outcomes; (4) used randomized controlled trial (RCT) or quasi‐experimental designs and achieved at least 60% of the total score on the relevant critical appraisal tools (e.g., a minimum score of 8 out of 13 for RCTs or 6 out of 9 for quasi‐experimental studies), indicating sufficient methodological quality to support evidence utilization; (5) clearly described the components and implementation details of hypertension management interventions; (6) reported relevant statistical outcomes related to hypertension management, such as knowledge, health behaviors, coping behavior/anxiety, awareness, blood pressure, or biomarkers; and (7) were published in English or Thai in peer‐reviewed journals, theses, or dissertations. However, older adults with mild cognitive impairment or depression were excluded from the study, as participants in the hypertension management program needed sufficient cognitive ability to engage in and benefit from the learning process.

### 3.4. Critical Appraisal and Grading

Evidence levels were indicated using the hierarchical evidence pyramid from the JBI [23]. Before including the research articles, the selected studies’ quality was assessed using the critical appraisal tool from JBI; the Checklists for RCT and Quasi‐Experimental Studies were applied for this review [24, 25]. Moreover, grading was provided for each study, which was included in the table of result reports [26]. The selected studies were required to meet a positive response (i.e., “yes”) on a minimum of six out of nine for quasi‐experimental studies and eight out of 13 for RCT [24, 25]. Two reviewers independently assessed the risk‐of‐bias using grading, and a risk‐of‐bias table was designed for each eligible study. Disagreements between reviewers were resolved by mutual consensus and the third reviewer. Methodological quality was categorized into very low–, low‐, moderate‐, and high‐quality categories [25]. Critical appraisal results were also reported in narrative form and a table. All selected studies reported their methodological quality and underwent data extraction and synthesis.

### 3.5. Study Selection and Data Extraction

This process involved two main steps: study selection and data extraction. During the study selection phase, two reviewers independently assessed retrieved records for eligibility to minimize selection bias. Studies were screened in two sequential stages. First, titles and abstracts were reviewed to identify potentially relevant studies regarding the hypertension management program among community‐dwelling older adults. Second, the full texts of the remaining articles were assessed against the predefined inclusion and exclusion criteria. Any disagreements between reviewers regarding eligibility were resolved through discussion and consensus, with a third reviewer consulted when necessary.

The study identification process followed the Preferred Reporting Items for Systematic Reviews and Meta‐Analyses (PRISMA) guidelines. Records were identified through database searches, and duplicate records were removed prior to screening using reference management software. All screening decisions, including inclusion and exclusion at each stage, were documented in Rayyan, a reference management software, to ensure transparency and reproducibility. The number of records identified, screened, excluded, and included at each stage is presented in the PRISMA flow diagram, which also specifies the reasons for exclusion during the full‐text review stage.

Following study selection, data extraction was conducted. Before commencing extraction, a codebook and standardized data extraction forms were developed to ensure consistency. The extraction form captured key information from each included study, including (1) the studies’ authors, (2) study designs and aim, (3) levels of evidence/critical appraisal score/grading, (4) settings and population, (5) intervention, (6) content outline, (7) times of outcomes measuring, (8) selected outcomes of the hypertension management interventions, (9) results, and (10) research notes. The codebook and extraction form were pilot‐tested across five studies and refined accordingly before full extraction. After that, two reviewers independently extracted data from all included studies. Any discrepancies between reviewers were resolved through discussion, and when consensus could not be reached, a third reviewer was consulted. This process ensured accuracy, completeness, and methodological rigor in the extraction of study data.

## 4. Data Analysis

Evidence was synthesized using structured evidence tables. An extraction table was developed to present the key characteristics and findings of each included study, while an intervention component table summarized the intervention elements and delivery strategies. This approach facilitated the identification of recurring program structures and enabled the examination of how specific combinations of intervention components were associated with improvements in blood pressure outcomes.

Given the substantial clinical and methodological heterogeneity across the included trials—including differences in intervention intensity, cointerventions, blood pressure measurement schedules, analytical approaches, and reporting formats—a meta‐analysis was not considered appropriate. Instead, the findings were synthesized using structured quantitative narrative approaches.

## 5. Quantitative Synthesis and Heterogeneity Assessment

To provide quantitative interpretation in the absence of statistical pooling, blood pressure outcomes were summarized using two complementary approaches. First, vote counting based on the direction of effect was conducted to classify whether interventions were associated with improvement, no change, or worsening of systolic blood pressure (SBP) and diastolic blood pressure (DBP). Second, a quantitative narrative synthesis was performed by summarizing the median and range of blood pressure reductions across studies using reported baseline and postintervention mean values where available.

The included interventions varied substantially in their composition and delivery, incorporating different combinations of lifestyle modification components (e.g., dietary modification, physical activity promotion, stress management, and substance use reduction) and behavioral strategies (e.g., medication adherence support, self‐monitoring, and social support) delivered through approaches such as education, consultation, and monitoring. Follow‐up durations, comparator conditions, and outcome reporting formats also differed across studies. Consistent with the objective of this review—to characterize the intervention components and delivery strategies of hypertension management programs—this narrative quantitative synthesis was considered the most appropriate method for summarizing the evidence.

## 6. Validity, Reliability, and Rigor

A research team comprising individuals two researchers and expertise levels conducted this investigation. Collaboratively, systematic review experts and librarians collaborated to formulate a search statement, select essential databases related to the study topic, and search for articles together. The team iteratively refined the systematic review protocol and search statement. The principal investigator prospectively registered a study protocol before conducting this systematic review, adhering rigorously to the protocol to mitigate bias and enhance the study’s validity and reliability. A third independent researcher provided a third opinion to resolve disagreements when discrepancies occurred. Finally, we contacted the three principal investigators to find the full paper and missing information. Fortunately, all requisite data were obtained, and we can provide completed interventions and results for this systematic review.

## 7. Results

### 7.1. Study Selection

**FIGURE 1:**
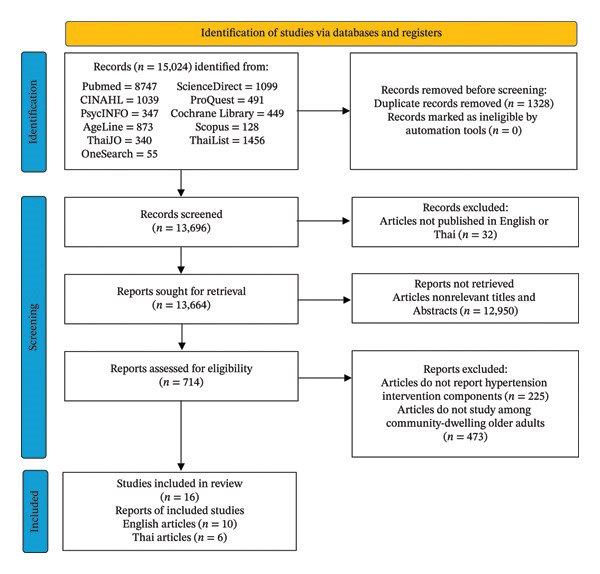
PRISMA flowchart of review process results.

From the initial 15,024 articles identified, 1328 duplicates were removed, and the titles and abstracts of the remaining 13,696 articles were screened. Based on the eligibility criteria and consensus among the research team, 16 articles were selected for the final comprehensive review, standard critical appraisal, and synthesis. As a result, the PRISMA flow diagram of the information flow during the review process showed that a total of 16 studies met the inclusion criteria and were included in the final synthesis (Figure 1).

### 7.2. Characteristics of Included Studies

Publication years for the 16 articles ranged from 2019 to 2024. Two were in 2019 [27, 28], four were in 2020 [29–32], three were in 2021 [33–35], five were in 2022 [36–40], one was in 2023 [41], and one was in 2024 [42]. Most studies were conducted in Southeast Asia, particularly in Thailand [28, 31–33, 38–40], two from China [34, 42], and one each from Korea [27], Iran [29], Turkey [30], Hong Kong [36], Canada [37], Indonesia [35], and the United States [41]. All study participants were exclusively older adults, comprising 100% of the sample in each study. The total number of participants was 1,857, and the number of participants in the included studies ranged from 30 to 200 older adults. All sixteen articles indicated the participant’s sex, both male and female community‐dwelling older adults who have been diagnosed with hypertension. There were two articles that provided experimental interventions at the hospital on the first day and then continuing care in the community until the end of the program [29, 33].

### 7.3. Methodological Quality of Included Studies

Methodological quality was assessed using JBI critical appraisal tools, and studies were categorized as low, moderate, or high quality based on predefined criteria. As a result, scores for ten RCTs were nine to twelve out of a possible 13 points [27, 29, 30, 33, 34, 36–38, 41, 42] (Table 1). The critical appraisal scores of the six quasi‐experimental studies ranged from seven to nine out of a possible nine points [28, 31, 32, 35, 39, 40]. No studies were excluded based on the critical appraisal score criteria (Table 2). Sixteen articles were graded for the level of quality. Two of those had low methodological quality [31, 37], four had moderate methodological quality [33, 34, 36, 41], and ten had high methodological quality [27–30, 32, 35, 38–40, 42] (Table 3).

**TABLE 1:** Critical appraisal of the selected randomized controlled trials (RCTs).

JBI Critical Appraisal Checklist for Randomized Controlled Trials Tufanaru, C., Munn, Z., Aromataris, E., Campbell, J., Hopp, L. (2020). Chapter 3: Systematic reviews of effectiveness. In: Aromataris E, Munn Z (editors). JBI Manual for Evidence Synthesis. Available from https://synthesismanual.jbi.global														
Studies/total score	Was true randomization used for assignment of participants to treatment groups?	Was allocation to treatment groups concealed?	Were treatment groups similar at the baseline?	Were participants blind to treatment assignment?	Were those delivering treatment blind to treatment assignment?	Were outcomes assessors blind to treatment assignment?	Were treatment groups treated identically other than the intervention of interest?	Was follow‐up complete and if not, were differences between groups in terms of their follow‐up adequately described and analyzed?	Were participants analyzed in the groups to which they were randomized?	Were outcomes measured in the same way for treatment groups?	Were outcomes measured in a reliable way?	Was appropriate statistical analysis used?	Was the trial design appropriate, and any deviations from the standard RCT design (individual randomization, parallel groups) accounted for in the conduct and analysis of the trial?	Total (13)
Kim [27]	Y	Y	Y	U	Y	Y	Y	Y	Y	Y	Y	Y	Y	12
Delavar et al. [29]	Y	Y	Y	U	U	U	Y	Y	Y	Y	Y	Y	Y	10
Kolcu and Ergun [30]	Y	Y	Y	U	N	U	Y	Y	Y	Y	Y	Y	Y	10
Audthiya et al. [33]	Y	Y	Y	N	N	Y	Y	Y	Y	Y	Y	Y	Y	11
Zhang et al. [34]	Y	Y	Y	Y	Y	Y	Y	Y	Y	Y	U	Y	Y	12
Wong et al. [36]	Y	Y	Y	Y	Y	Y	Y	Y	Y	Y	U	Y	Y	12
Lau et al. [37]	Y	Y	Y	N	N	Y	Y	Y	Y	Y	Y	Y	Y	11
Ongkulna et al. [38]	Y	Y	Y	N	N	N	Y	Y	Y	Y	Y	Y	Y	10
Kohn et al. [41]	Y	Y	Y	N	N	N	Y	Y	Y	Y	U	Y	Y	9
Sun et al. [42]	Y	Y	Y	U	Y	Y	Y	Y	Y	Y	Y	Y	Y	12

*Note:* Y = Yes; N = No; U = Unclear.

**TABLE 2:** Critical appraisal of the selected quasi‐experimental studies.

JBI critical appraisal checklist for quasi‐experimental studies. Tufanaru, C., Munn, Z., Aromataris, E., Campbell, J., Hopp, L. (2020). Chapter 3: Systematic reviews of effectiveness. In: Aromataris E, Munn Z (Editors). JBI Manual for Evidence Synthesis. Available from https://synthesismanual.jbi.global										
Studies/total score	Is it clear in the study what is the cause and what is the effect (i.e., there is no confusion about which variable comes first)?	Were the participants included in any comparisons similar?	Were the participants included in any comparisons receiving similar treatment/care, other than the exposure or intervention of interest?	Was there a control group?	Were there multiple measurements of the outcome both pre and postintervention/exposure?	Was follow‐up complete and if not, were differences between groups in terms of their follow‐up adequately described and analyzed?	Were the outcomes of participants included in any comparisons measured in the same way?	Were outcomes measured in a reliable way?	Was appropriate statistical analysis used?	Total (9)
Anantasaran [28]	Y	Y	Y	Y	Y	Y	Y	Y	Y	9
Kitrungrote [31]	Y	Y	Y	N	Y	N	Y	Y	Y	7
Woodham et al. [32]	Y	Y	Y	Y	Y	Y	Y	Y	Y	9
Putri et al. [35]	Y	Y	Y	Y	Y	N	Y	Y	Y	8
Sukpattanasrikul et al. [39]	Y	Y	Y	Y	Y	N	Y	Y	Y	8
Bumrungsuk [40]	Y	Y	Y	Y	Y	N	Y	Y	Y	8

*Note:* Y = Yes; N = No; U = Unclear.

**TABLE 3:** Quality assessment results of the selected studies by grade guideline.

Quality assessment of the evidence by grade guideline Schünemann H, Brożek J, Guyatt G, Oxman A, editors. GRADE Handbook. 2013. Available from: https://gdt.gradepro.org/app/handbook/handbook.html?fbclid%3dIwAR04O97yy.										
Studies	^a^Risk of bias (limitation of study design, confounding factors, missing data, adherence measurement)			^b^Precision (methodology, statistical certainty, amount of information on a certain factor how precisely an object of study is measured)		^c^Directness (extent to which the people, interventions, and outcome measures are similar to those of interest, confident results come from the direct evidence)		^d^Consistency (relevant measurement application where several items that propose to measure the same general construct produce similar scores, no overlapping and missing, statistical significance)		Certainty of evidence
	Low	Unclear	High	Precise	Imprecise	Direct	Indirect	Consistent	Inconsistent	
Anantasaran [28]	✓			✓		✓		✓		High
Kim [27]	✓			✓		✓		✓		High
Delavar et al. [29]	✓			✓		✓		✓		High
Kolcu and Ergun [30]	✓			✓		✓		✓		High
Kitrungrote [31]			✓	✓		✓			✓	Low
Woodham et al. [32]	✓			✓		✓		✓		High
Audthiya et al. [33]	✓			✓		✓			✓	Moderate
Putri et al. [35]	✓			✓		✓		✓		High
Zhang et al. [34]	✓				✓	✓		✓		Moderate
Sukpattanasrikul et al. [39]	✓			✓		✓		✓		High
Wong et al. [36]	✓				✓	✓		✓		Moderate
Bumrungsuk [40]	✓			✓		✓		✓		High
Lau et al. [37]	✓				✓		✓	✓		Low
Ongkulna et al. [38]	✓			✓		✓		✓		High
Kohn et al. [41]		✓		✓		✓		✓		Moderate
Sun et al. [42]	✓			✓		✓		✓		High

^a^Risk of bias.

^b^Precision.

^c^Directness.

^d^Consistency.

### 7.4. Hypertension Management Program for Community‐Dwelling Older Adults

#### 7.4.1. Nutrition Promotion

Nutrition promotion emerged as a vital component of hypertension management, aiming to improve dietary habits and reduce blood pressure through targeted nutritional guidance. Nutritional promotions were found in 10 articles that mentioned integrating nutritional education sessions within a self‐care behavior‐promoting program, emphasizing the importance of balanced diets with unspecific food consumption, portion control, and reduced sodium intake [28, 30–32, 35, 38–42]. Phone‐based health coaching provides personalized nutritional advice, focusing on maintaining a heart‐healthy diet. These programs included skill‐training workshops to help participants identify healthier food choices, understand food labels, and implement dietary changes that align with hypertension management guidelines. Nutritional counseling was often complemented by follow‐up support through home visits or telephone check‐ins to reinforce dietary changes and address any challenges faced by the participants. This counseling would help to manage and control blood pressure and help delay or prevent possible life‐threatening complications (Table 4).

**TABLE 4:** A summary of the reviewed studies about effectiveness of hypertension management interventions among community‐dwelling older adults.

Authors/country	Designs: aims	Level of evidence/critical appraisal/grading	Setting/population	Interventions: ways of distributing intervention	Content outline	Time to measure	Selected outcomes: tool	Results	Notes
Anantasaran [28]/Thai	Quasi: To evaluate the effectiveness of a self‐care behavior –promoting program on improving self‐care behaviors and reducing blood pressure levels among older adults with uncontrolled hypertension in Langsuan municipality, Chumphon province.	Level 2.c 9/9 High	Community/Each group was no difference significance. (No details) ‐ Most participants in both groups were female, accounting for 81.67% of the total participants. ‐ Intervention group average age was 68.01 years. Min 61, Max 71 ‐ Control group average age was 67.33 years. Min 62, max 74 ‐ Older adult 100%	‐ Delivered a 10‐week self‐care behavior‐promoting program for older adults with hypertension. ‐ Conducted two three‐hour education sessions on hypertension, risks, and lifestyle modification. ‐ Provided skill‐training workshops to improve self‐care and prevent complications. ‐ Conducted home visits for personalized guidance and adherence support. ‐ Implemented weekly follow‐up calls to monitor progress and maintain motivation.	‐ Nutritional guidance. ‐ Physical activity training. ‐ Stress management techniques. ‐ Home visit for personalized support. ‐ Weekly telephone follow‐up.	‐ Before intervention. ‐ 10 weeks after intervention.	‐ Systolic blood pressure. ‐ Diastolic blood pressure. ‐ Hypertension knowledge. ‐ Perceive risk. ‐ Perceive severity. ‐ Perceive benefits. ‐ Self‐care behaviors. (Complication prevention)	Results of pretest–post‐test of the intervention/control group. ‐ Systolic blood pressure 154.50 ± 10.11, 140.11 ± 10.57/153.78 ± 9.87, 148.96 ± 10.23 *p* value 0.001 ‐ Diastolic blood pressure 99.82 ± 11.79, 89.75 ± 10.85/98.85 ± 11.78, 95.33 ± 0.58 *p* value 0.005 ‐ Knowledge 7.7 ± 1.0, 8.9 ± 1.3/7.4 ± 1.2, 7.8 ± 2.0 *p* value < 0.001 ‐ Perceive risk 9.7 ± 2.4, 11.6 ± 2.8/8.9 ± 3.0, 9.1 ± 2.9 *p* value 0.001 ‐ Perceive severity 8.8 ± 2.4, 10.7 ± 2.8/7.8 ± 2.9, 8.1 ± 3.1 *p* value < 0.01 ‐ Perceive benefit 7.7 ± 2.4, 8.9 ± 2.1/6.9 ± 2.8, 7.6 ± 3.4 *p* value 0.127 ‐ Self‐behavior 10.7 ± 3.57, 14.5 ± 3.33/10.6 ± 5.74, 11.4 ± 4.57 *p* value 0.001	‐ No logbook to be evidence recorded to ensure that participants really follow the interventions activities. ‐ Telephone follow‐up is effective. However, encourage, progress, or other support will be more effective if there were the two‐way contact.

Kim [27]/Korea	RCT: To develop long‐message services (LMSs) and phone‐based health‐coaching for community‐dwelling seniors diagnosed with hypertension and evaluate the effects of these programs used separately and together.	Level 1.c 12/13 High	Senior welfare center./ ‐ Long‐message service (LMS) total 32 people, male 12 (37.5%), female 20 (62.5%), mean age 77.21 ± 6.69. ‐ Health‐coaching with LMS total 31 people, male 17 (54.8%), female 14 (45.2%), mean age 78.25 ± 6.61. ‐ Control total 31 people, male 20 (64.5%), female 11 (35.5%), mean age 77.70 ± 6.92. ‐ Older adult 100%	‐ Phone‐based health coaching: Provided personalized sessions to enhance medication adherence, self‐efficacy, self‐management behaviors, and reduce systolic blood pressure. ‐ Long‐message service (LMS): Delivered tailored educational messages to strengthen hypertension knowledge and self‐care practices. ‐ Combined coaching with LMS: Integrated personalized phone coaching with LMS education, offering both interactive guidance and continuous learning support.	‐ Definition of hypertension and complications. ‐ Healthy diet. ‐ Taking medicine. ‐ Regular exercise. ‐ Weight control. ‐ Stress management. ‐ Nonsmoking and non‐drinking. ‐ Healthy sleep.	‐ 1 week after the intervention. ‐ 8 weeks after the intervention.	‐ Medication adherence. ‐ Self‐efficacy. ‐ Self‐management behaviors. ‐ Systolic blood pressure. ‐ Hypertension Knowledge.	Each outcome of control, health‐coaching, long‐message service, and health‐coaching with LMS there are ‐ Medication adherence 0.87 ± 1.08, 2.50 ± 1.22, 1.87 ± 1.38, and 2.87 ± 1.11 respectively. *p* value < 0.001 ‐ Self‐efficacy 7.77 ± 0.90, 8.34 ± 0.81, 7.52 ± 0.78 , and 8.68 ± 0.69, respectively. *p* value < 0.001 ‐ Self‐management behaviors 61.93 ± 7.01, 71.80 ± 3.91 , 67.21 ± 5.77, and 73.00 ± 4.25, respectively. *p* value < 0.001 ‐ Systolic blood pressure 143.58 ± 9.49, 132.53 ± 8.92 , 139.15 ± 7.51 , and 133.61 ± 7.32, respectively. *p* value < 0.001 ‐ Hypertension knowledge 7.77 ± 1.54, 8.63 ± 0.92 , 8.59 ± 0.97 , and 9.45 ± 0.88 *p* value < 0.001	‐ Long‐Message Service may be ineffective for the older adults who has degenerative progression of the physical, should contain more media, therefore video, flex message, flex card, voice, etc.

Delavar et al. [29]/Iran	RCT: To evaluate the effects of self‐management education tailored to health literacy on medication adherence and blood pressure control among older adults individuals with uncontrolled primary hypertension and inadequate health literacy.	Level 1.c 10/13 High	Mixed setting (Hospital and community)/ ‐ Intervention group total 56 people, male 23 (41.6%), female 31 (57.4%), mean age 64.0. (No S.D. report) ‐ Control group total 58 people, male 32 (55.2%), female 26 (44.8%), mean age 66.88 (No S.D. report) ‐ Older adult 100%	‐ Delivered a self‐management education program tailored to participants’ health literacy levels. ‐ Covered key topics including hypertension definition, risk factors, complications, medication use and side effects, adherence importance, and regular blood pressure monitoring. ‐ Conducted two face‐to‐face weekly sessions (30–45 min each) followed by four telephone sessions held twice weekly (15 min each). ‐ Applied the teach‐back method in both in‐person and telephone sessions to reinforce understanding and retention.	‐ Hypertension definition ‐ Risk factors ‐ Complications ‐ Medications ‐ Medication side effects ‐ Medication adherence importance ‐ Importance of regular blood pressure monitoring.	‐ Before intervention. ‐ 1 month after intervention. ‐ 3 months after intervention.	‐ Medication adherence: Morisky Medication Adherence Scale. ‐ Systolic blood pressure. ‐ Diastolic blood pressure.	‐ Medication adherence and posttest in the intervention group (n = 56) were distributed as follows: poor adherence in 27 participants (50.0%), moderate adherence in 26 participants (48.1%), and good adherence in 1 participant (1.9%) In the control group (n = 58), posttest revealed poor adherence in 46 participants (79.3%), moderate adherence in 12 participants (20.7%), and good adherence in none (0.0%). A statistically significant difference in medication adherence was observed between the two groups (*p* = 0.002) ‐ Systolic blood pressure between group on post‐test was 142.59 ± 11.56, 148.45 ± 12.32 *p* value 0.011, within‐intervention group 156.43 ± 12.38, 142.59 ± 11.56 *p* value < 0.001 ‐ Diastolic blood pressure between group on post‐test was 88.52 ± 7.99, 92.15 ± 9.33 *p* value 0.029, within‐intervention group 96.11 ± 6.85, 88.52 ± 7.99 *p* value < 0.001	‐ Extend the study duration to include long‐term follow‐ups to assess the sustained effects. ‐ Utilizing medication adherence. ‐ Collaborate with participant to cocreate the educational materials and intervention strategies, ensuring that they are tailored to their specific health literacy needs and preferences.

Kolcu and Ergun [30]/Turkey	RCT: To evaluate the effects of a nurse‐led hypertension management program on quality of life, medication adherence and hypertension management in older adults.	Level 1.c 10/13 High	Community (Nursing home)/ ‐ Intervention group total 37 people, male 19 (51.4%), female 18 (48.6%), age 65–74 total 18 people 48.6%, above 74 total 19 people 51.4%. ‐ Control group total 37 people, male 21 (56.8%), female 16 (43.2%), age 65 – 74 total 16 people 43.2%, above 74 total 21 people 56.9%. ‐ Older adult 100%	‐ Received tailored care through the NLHMP, which involved education, monitoring: group education sessions and individual education. ‐ Activities within the NLHMP encompassed clinical evaluations every 6 months, procurement and administration of antihypertensive medications, and regular blood pressure monitoring twice a day. ‐ Participants in the NLHMP exhibited lower blood pressure and cholesterol levels after intervention.	‐ Health promoting lifestyle changes. ‐ Medication adherence training. ‐ Blood pressure monitoring guidance. ‐ Nutrition counseling. ‐ Physical activity. ‐ Stress management techniques.	‐ Before intervention. ‐ Week 1 after intervention. ‐ Week 2 after intervention. ‐ Week 5 after intervention. ‐ Week 24 after intervention.	‐ Systolic blood pressure. ‐ Diastolic blood pressure. ‐ Hypertension knowledge. ‐ Medication adherence: MMAS‐4 ‐ Body mass index. ‐ Quality of life.	Intervention group (pretest, post‐test); control group (pretest, post‐test) ‐ Systolic blood pressure 129.18 ± 14.60, 118.64 ± 10.04 *p* value 0.001; 119.18 ± 15.16, 130.54 ± 15.08 post‐test between group *p* value < 0.001 ‐ Diastolic blood pressure 79.72 ± 9.57, 77.83 ± 5.34 *p* value 0.903; 75.13 ± 10.17, 82.70 ± 7.69 post‐test between group *p* value 0.490 ‐ Knowledge score 12.10 ± 1.92, 20.75 ± 1.01 *p* value < 0.001; 12.02 ± 2.15, 12.21 ± 2.48 post‐test between group *p* value < 0.001 ‐ Medication adherence Intervention/Control pretest; intervention/control post‐test (high, moderate, low) 30 (81.1%), 6 (16.2), 1 (2.7)/24 (64.9%), 8 (21.6%), 5 (13.5%) *p* value 0.164; 37 (100.0%), 0 (0.0%), 0 (0.0%)/24 (64.9%), 8 (21.6%), 5 (13.5%) *p* value < 0.001 ‐ Body mass index 28.13 ± 4.96, 27.19 ± 4.92 *p* value < 0.001; 26.59 ± 4.87, 27.22 ± 4.78 post‐test between group *p* value < 0.001 ‐ Quality of life (SF‐36) has no significant change within group in terms of pretest but post‐test in intervention group were significant higher at *p* value < 0.05 there were physical functioning, role‐physical, pain, general health, vitality, social functioning, role‐emotional, mental health, physical component, and mental component.	‐ Incorporate personalized health‐coaching sessions where older adults receive tailored advice and support based on their individual health goals and preferences, promoting a sense of autonomy and empowerment in managing their hypertension. ‐ Implement hypertension management workshops or support groups, to foster social connections among older adults and create a supportive environment for sustainable lifestyle changes.

Kitrungrote [31]/Thai	Quasi: To evaluate the effects of a nutritional education support program on dietary behaviors and blood pressure level in older adults’ club members with uncontrolled hypertension.	Level 2.d 7/9 Low	Community/ ‐ Intervention group total 36 people, male 8 (22.2%), female 28 (77.8%), mean age 70.81 ± 8.35 (Min 60, max 94) ‐ Older adult 100%	‐ Weeks 1–8: Monitored blood pressure and provided health education integrated with praying and meditation activities. ‐ Weeks 8–15: Divided participants into six small groups of six members each, meeting five times for 1–1.5 h per session. ‐ Weeks 8, 9, and 11: conducted group discussions on healthy dietary habits following the DASH diet. ‐ Weeks 12–14: Focused on the practical application of the DASH diet, with participants recording dietary behaviors and blood pressure in personal logbooks.	‐ Experiential learning. ‐ DASH‐diet information.	‐ Before intervention. ‐ 1 week after intervention. ‐ 8 weeks after intervention. ‐ 15 weeks after intervention.	‐ Dietary behaviors. ‐ Systolic blood pressure. ‐ Diastolic blood pressure.	‐ Dietary behaviors 1.91 ± 0.91, 2.25 ± 0.30 *p* value < 0.001. ‐ Systolic blood pressure Week 1 and Week 15. There is a difference in averages. −14.00 (*p* < 0.001) Week 18 and Week 15. There is a difference in averages −14.72 (*p* < 0.001) ‐ Diastolic blood pressure Week 1 and Week 15. There is a difference in averages. −8.22 (*p* < 0.01) Week 18 and Week 15. There is a difference in averages −6.11 (*p* < 0.01)	‐ No detail of mean, S.D. in each period, there was only final one‐way‐repeated ANOVA table. ‐ No compare groups.

Woodham et al. [32]/Thai	Quasi: To examining the effectiveness of multidisciplinary approach intervention to enhance blood pressure control among older adults hypertensive patients in rural Thailand	Level 2.c 9/9 Moderate	Community/ ‐ Intervention group total 100 people, male 26 (26.0%), female 74 (74.0%), mean age 67.49 ± 6.15. ‐ Control group total 100 people, male 29 (29.0%), female 71 (71.0%), mean age 66.88 ± 5.70. ‐ Older adult 100%	‐ Healthcare teams provided continuous monitoring and community‐based support to help older adults manage blood pressure effectively. ‐ Family members actively participated in the care process to strengthen adherence and emotional support in hypertension management. ‐ Delivered in‐person education sessions with pamphlets and brochures emphasizing the importance of regular and correct antihypertensive medication use. ‐ Implemented electronic pill boxes with visual and auditory alerts. ‐ Conducted monthly pill counts to monitor medication adherence and track progress in blood pressure control.	‐ Medication adherence. (Importance of taking medications, Instruction on the correct dosage, timing, and potential side effects) ‐ Lifestyle modification. (Regular exercise, balanced diet, and stress management)	‐ Before intervention. ‐ 1 month after intervention. ‐ 3 months after the intervention.	‐ Systolic blood pressure. ‐ Diastolic blood pressure. ‐ Medication adherence. ‐ eGFR.	Each outcome of the baseline, 1‐month, and 3‐months after intervention were as follows: Intervention group ‐ SBP: 154.51 ± 11.16, 143.29 ± 12.58, and 141.27 ± 13.59, respectively. *p* value < 0.001. ‐ DBP: 90.47 ± 6.13, 82.59 ± 8.01, and 73.22 ± 8.97, respectively. *p* value 0.001. ‐ Medical adherence: 53.63 ± 17.57, 71.71 ± 18.49, and 71.40 ± 16.06, respectively. *p* value < 0.001. ‐ eGFR: 90.66 ± 17.97, and 96.75 ± 13.91 *p* value < 0.001.	‐ Nutritional counseling and cooking workshops, offer personalized to educate nutrition knowledge and provide practical skills for preparing DASH diet. ‐ Establish support groups where participants can share experiences, challenges, and successes related to blood pressure management.

Audthiya et al. [33]/Thai	RCT: To evaluate the effectiveness of a patient‐centered communication program in enhancing autonomy and self‐management behaviors among older adults with hypertension in Thailand	Level 1.c 11/13 Moderate	Hospital/ ‐ Intervention group total 30 people, male 16 (53.3%), female 14 (46.7%), mean age 68.73 ± 4.14 ‐ Control group total 30 people, male 15 (50.0%), female 15 (50.0%), mean age 69.48 ± 4.97 ‐ Older adult 100%	‐ Provided communication training to enhance interactions with physicians and support information exchange. ‐ Conducted individual assessments to identify needs, set goals, and create personalized self‐management plans. ‐ Delivered skill‐based sessions focused on knowledge building, problem‐solving, and overcoming barriers. ‐ Offered telephone follow‐ups and counseling to reinforce learning, motivation, and sustained behavior change.	‐ Education on hypertension management, treatment options, and lifestyle modification. ‐ Offered psychological support to address emotional concerns and enhance self‐care confidence.	‐ Before intervention. ‐ 12 weeks after intervention. ‐ 12 weeks after intervention finished.	‐ Autonomy. ‐ Self‐management behaviors.	Result at baseline, at the program end, and 3 months after intervention of the intervention group/control group. ‐ Autonomy 37.43 ± 6.85, 56.37 ± 4.52, 57.77 ± 3.71/40.57 ± 6.41, 39.90 ± 5.20, 42.10 ± 5.63 *p* value 0.072, < 0.001, < 0.001, respectively. ‐ Self‐management behaviors 69.77 ± 12.57, 92.67 ± 11.33, 102.73 ± 8.79/69.07 ± 9.15, 67.93 ± 8.03, 67.60 ± 9.17 *p* value 0.806, < 0.001, < 0.001, respectively.	‐ Did not study the change of the blood pressure, although referred to the hypertension patients. ‐ Incorporate gamified into educational materials and self‐care activities to make learning and adherence more engaging and motivating. ‐ Conduct support groups.

Putri et al. [35]/Indonesia	Quasi: To evaluate the effectiveness of self‐management on adherence to caring for themselves and on health status among older people with hypertension.	Level 2.c 8/9 High	Community/ ‐ Intervention group total 67 people, male: 20 (29.85%), female: 47 (70.15%), 60–74 years old: 55 (82.9%), 64–90 years old: 12 (17.91%) ‐ Control group total 67 people, male: 23 (34.33%), female: 44 (65.67%), 60–74 years old: 58 (86.57%), 64–90 years old: 9 (13.43%) ‐ Older adult 100%	‐ Implemented a self‐management program providing education and personalized guidance through home visits tailored to individual needs. ‐ Organized interactive group sessions featuring discussions and practical learning to enhance self‐care and adherence. ‐ Distributed educational modules and workbooks to reinforce hypertension self‐management skills. ‐ Ensured continuity of support through follow‐up by community health nurses and additional home visits to sustain behavioral changes.	‐ Understanding hypertension. ‐ Physical activities management and social support. ‐ Nutrition management. ‐ Relaxation techniques and Lifestyle change. ‐ Educational manual and workbook.	‐ Before intervention. ‐ 2 weeks after intervention.	‐ Adherence in self‐care practice (medical adherence, physical activity, dietary management) ‐ Self‐management health status	After the intervention shows a significantly higher score in intervention group (intervention, control groups) ‐ Adherence of self‐care 84.30 ± 9.05, 75.70 ± 6.29 *p* value < 0.001 ‐ Health status 49.31 ± 6.08, 41.39 ± 4.76 *p* value < 0.001	‐ Develop customized educational programs tailored individually. ‐ Encourage involvement of family members or caregivers to support, reinforce, and assist in adherence. ‐ Should implement long‐term follow‐up.

Sukpattanasrikul et al. [39]/Thai	Quasi: To evaluate the effects of a self‐management program (SMP) on self‐care behavior, blood pressure, and quality of life among older adults with uncontrolled hypertension.	Level 2.c 8/9 High	Community/ ‐ Intervention group total 76 people, mean age 70.00 ± 6.20 ‐ Control group total 76 people, mean age 71.40 ± 7.30 ‐ Older adult 100%	‐ Conducted a self‐management program with education, goal setting, and self‐monitoring. ‐ Provided personalized guidance and skill‐building on diet, exercise, stress control, and lifestyle change. ‐ Facilitated group discussions for shared learning and support. ‐ Continued Weeks 5–16 follow‐up with progress checks, reinforcement, and ongoing education by healthcare staff.	‐ Goal setting. ‐ Self‐monitoring. ‐ Medication adherence. ‐ Nutrition. ‐ Exercise. ‐ Stress management. ‐ Lifestyle modification. ‐ Ongoing education and regular health check.	‐ Before intervention. ‐ 4 weeks after intervention. ‐ 8 weeks after intervention. ‐ 12 weeks after intervention. ‐ 16 weeks after the intervention finished.	‐ Self‐Care Behavior for Older Adults with Hypertension Questionnaire: (SCBOAHQ) CVI 0.80 and Cronbach’s alpha of 0.92. ‐ World Health Organization Quality of Life‐BREF THAI: (WHOQOL‐BREF THAI) ‐ Systolic blood pressure. ‐ Diastolic blood pressure.	Result at baseline, at the program end, and 4 months of follow‐up of the intervention/control group. ‐ Self‐care behavior 100.10 ± 11.77, 130.35 ± 7.79, 133.29 ± 8.43/103.23 ± 13.03, 102.01 ± 13.59, 101.13 ± 10.92 *p* value < 0.001 ‐ Diet control 40.03 ± 4.65, 52.79 ± 3.00, 51.95 ± 3.30/41.24 ± 4.49, 40.49 ± 4.65, 40.39 ± 4.14 *p* value < 0.001 ‐ Exercise control 16.37 ± 7.99, 29.01 ± 6.16, 31.61 ± 5.80/17.86 ± 7.66, 17.51 ± 8.10, 16.97 ± 7.46 *p* value < 0.001 ‐ Stress management 22.77 ± 2.70, 26.03 ± 1.63, 26.59 ± 1.32/23.27 ± 2.61, 23.01 ± 2.02, 23.29 ± 2.34 *p* value < 0.001 ‐ Taking medication 20.94 ± 1.45, 22.51 ± 0.92, 23.14 ± 0.76/20.85 ± 3.33, 21.00 ± 3.07, 20.48 ± 3.19 *p* value < 0.001 ‐ Systolic blood pressure was measure at baseline, 4^th^ weeks, 8^th^ weeks, 12^th^ weeks, 16^th^ weeks, respectively. Intervention group 150.23 ± 7.10, 136.05 ± 11.34, 133.05 ± 9.87, 133.93 ± 10.64, 133.91 ± 3.30/control group 150.88 ± 6.22, 149.64 ± 7.21, 150.00 ± 9.89, 150.05 ± 9.18, 149.17 ± 9.17 *p* value < 0.001 ‐ Diastolic blood pressure Intervention group 81.91 ± 9.36, 80.01 ± 9.20, 77.21 ± 8.37, 78.21 ± 9.48, 77.03 ± 9.21/control group 82.96 ± 9.47, 82.13 ± 8.65, 82.73 ± 0.35, 83.51 ± 8.65, 86.35 ± 9.44 *p* value < 0.001	‐ Provide mobile applications or wearable devices to monitor blood pressure, track medication adherence, and provide real‐time feedback to participants. ‐ Gamify of the self‐management program to make it more engaging and enjoyable for older adults. ‐ Establish support groups where older adults with uncontrolled hypertension can connect, share experiences. ‐ Provide educational materials, training sessions, and resources for family members to enhance their support of older adults.

Zhang et al. [34]/China	RCT: To evaluate the effects of the RAM applied to hypertension nursing on the self‐management behavior of older adult patients, their medication compliance, quality of life, and blood pressure control effect	Level 1.c 12/13 Moderate	Mixed setting (hospital and community)/ ‐ Intervention group total 60 people, male 33 (55.0%), female 27 (45.0%), mean age 74.22 ± 7.97 ‐ Control group total 60 people, male 32 (53.3%), female 28 (46.7%), mean age 75.27 ± 81.2 ‐ Older adult 100%	‐ Applied the Roy adaptation model (RAM) nursing approach to strengthen patients’ adaptability. ‐ Addressed physiological function, interdependence, role function, and self‐concept. ‐ Enhanced physiological function through comfortable environments, standardized blood pressure monitoring, and self‐care guidance. Promoted interdependence by encouraging family involvement, confidence building, and social connection. Reinforced role function to increase patients’ responsibility and engagement in care. Supported self‐concept through active communication, diverse educational media, group support, and coping strategy development.	‐ RAM nursing procedures. ‐ Physiological function. ‐ Family support ‐ Empowerment. ‐ Self‐concept: communication, diverse health education methods (health manuals, videos, WeChat posts, and group support.	‐ Before intervention. ‐ 12 weeks after intervention.	‐ Self‐efficacy. ‐ Self‐management. ‐ Medication compliance. ‐ Quality of life. ‐ Systolic blood pressure. ‐ Diastolic blood pressure.	After the intervention shows a significantly higher score in intervention group (intervention, control groups) ‐ Self‐efficacy total score 40.96 ± 3.72, 36.92 ± 4.88 *p* value < 0.001 ‐ Self‐management total score 148.02 ± 11.44, 124.04 ± 15.04 *p* value < 0.001 ‐ Medication compliance 6.57 ± 1.47, 4.90 ± 2.16 *p* value < 0.001 ‐ Quality of life total score 642.46 ± 75.13, 553.05 ± 111.20 *p* value < 0.001 ‐ Systolic blood pressure 135.77 ± 7.56, 142.50 ± 11.06 *p* value 0.001 ‐ Diastolic blood pressure 73.81 ± 6.50, 77.65 ± 8.81 *p* value 0.017	‐ Utilize telemedicine to provide remote the monitoring of blood pressure, medication adherence, and lifestyle behaviors. ‐ Implement of wearable devices and mobile applications to track blood pressure, physical activity, and dietary patterns.

Wong et al. [36]/Hong Kong	RCT: To evaluate the effects of an interactive mHealth program supported by a health‐social partnership team on quality of life (QOL) among community‐dwelling older adults in Hong Kong.	Level 1.c 12/13 High	Community/ ‐ mHealth + interactive group total 74 people, male 14 (18.9%), female 60 (81.1%), mean age 74.7 ± 7.6 ‐ mHealth group total 71 people, male 9 (12.7%), female 62 (87.3%), mean age 77.6 ± 7.84 ‐ Control group total 76 people, male 13 (17.1%), female 63 (82.9%), mean age 77.4 ± 8.2 ‐ Older adult 100%	‐ Implemented three groups: mHealth + I, mHealth, and control. ‐ The mHealth + I group received an interactive mobile health program with nurse support, offering personalized education and proactive follow‐up calls. ‐ Guided by Bandura’s self‐efficacy theory, the program used strategies such as recalling successful self‐care experiences and verbal encouragement to strengthen confidence and self‐management. Aimed to empower participants to actively manage their health and enhance self‐care abilities.	‐ The mHealth + I group received an interactive mHealth program with the support of a health‐social partnership team, including a nurse acting as a case manager. ‐ Proactive care, comprehensive health assessments, personalized health education, nurse support, and integrated health‐social team efforts to enhance the overall well‐being and quality of life.	‐ Before intervention. ‐ 3 months after intervention.	‐ Quality of life. ‐ Self‐efficacy score. ‐ Systolic blood pressure. ‐ Diastolic blood pressure.	Result of mHealth + I, mHealth, control group at baseline/Result of mHealth + I, mHealth, control group at program end. ‐ Quality of life (physical component summary) 38.75 ± 1.08, 39.98 ± 1.20, 39.76 ± 1.17/42.14 ± 1.04, 42.85 ± 1.25, 41.62 ± 0.93 *p* value < 0.001 ‐ Quality of life (mental component summary) 49.71 ± 1.25, 52.31 ± 1.29, 50.58 ± 1.32/49.51 ± 1.09, 50.4 ± 1.20, 49.67 ± 1.25 *p* value < 0.001 ‐ Self‐efficacy score 24.39 ± 0.68, 26.15 ± 0.81, 26.70 ± 0.73/26.78 ± 0.73, 27.73 ± 0.62, 26.28 ± 0.61 *p* value < 0.001 ‐ Systolic blood pressure 136.27 ± 2.09, 139.51 ± 0.81, 136.83 ± 2.73/132.95 ± 2.34, 137.13 ± 2.01, 137.81 ± 3.05 *p* value < 0.001 ‐ Diastolic blood pressure 71.02 ± 1.16, 71.99 ± 1.22, 71.72 ± 1.24/70.41 ± 1.58, 71.74 ± 1.79, 71.44 ± 1.56 *p* value < 0.001	‐ The paper does not provide detailed information on the recruitment process of participants, potentially affecting the generalize of findings. ‐ There is a lack of discussion on the potential biases or confounding variables that may have influenced the results.

Bumrungsuk [40]/Thai	Quasi: To evaluate the effects of the self‐management training program on self‐management behavior and blood pressure levels among older adults with hypertension	Level 2.c 8/9 High	Community/ ‐ Intervention group total 44 people, male: 8 (18.2%), female: 36 (81.8%), age < 55 years old: 6 (13.6%), 56–65 years old 18 (40.9%), 66–75 years old: 15 (34.1%), 76–85 years old 5 (11.4%) ‐ Control group total 47 people, male: 6 (12.8%), female: 41 (87.2%), age < 55 years old: 6 (12.8%), 56–65 years old: 26 (55.3%), 66–75 years old: 12 (25.5%), 76–85 years old: 3 (6.4%) ‐ Older adult 100%	‐ Self‐management training program included small‐group health‐education sessions and the distribution of the patient’s manual, Self‐Management Training Skills for Self‐Management Behavior. ‐ Participants engaged in small‐group discussions aimed at reflecting on self‐management concepts and behaviors, with a focus on stimulating follow‐up activities for self‐management behavior training during home visits. ‐ Received guidance on managing their blood pressure levels through regular assessments and tracking of their progress.	‐ The education sessions focused on causes, symptoms, complications, and treatments of hypertension, including the importance of proper nutrition for the older adults with HT. ‐ Small‐group discussion on food choices, exercise routines like the Dao De Xin Xi dance mixture, meditation, relaxation, and self‐management principles for effective BP control. ‐ Home visiting.	‐ Before intervention. ‐ 4 weeks after intervention. ‐ 13 weeks after intervention.	‐ Systolic blood pressure. ‐ Diastolic blood pressure. ‐ Self‐management behaviors.	Result of pretest–post‐test in the intervention/control group ‐ Systolic blood pressure 138.27 ± 18.57, 130.45 ± 26.30 *p* value 0.002/132.49 ± 15.93, 134.47 ± 14.31 *p* value 0.060. ‐ Diastolic blood pressure 73.75 ± 11.47, 78.91 ± 3.36/75.96 ± 9.63, 77.81 ± 11.35 *p* value 0.009 ‐ Self‐management behaviors at before, 4^th^ weeks, and 13^th^ weeks after the intervention of intervention/control group. 2.86 ± 0.448, 3.26 ± 0.197, 3.08 ± 0.375/2.66 ± 0.541, 2.67 ± 0.517, 2.64 ± 0.545 *p* values between group and within group were both < 0.001	‐ Lack of long‐term follow‐up to evaluate the sustained effectiveness of the Self‐Management Training Program. ‐ Should provide interactive workshops or support group, for enhancing motivation, adherence.

Lau et al. [37]/Canada	RCT: To compare the efficacy and safety of combining home blood pressure monitoring (HBPM) telemonitoring and protocolized case management with enhanced usual care using HBPM only in older community‐dwelling adults.	Level 1.c 11/13 Low	Community/ ‐ Intervention group total 61 people, male 19 (31.0%), female: 42 (69.0%), mean age: 79.8 ± 7.7 ‐ Control group total: 59 people, male: 9 (15.0%), female: 50 (85.0%), mean age: 79.2 ± 7.4 ‐ Older adult: 100%	‐ Equipped participants with wireless home blood pressure monitors enabling real‐time telemonitoring. ‐ Instructed to perform 7‐day blood pressure monitoring every 3 months, measuring twice daily and averaging readings for treatment adjustment. ‐ Pharmacists provided behavioral counseling, self‐monitoring education, and adherence support. ‐ Participants not achieving blood pressure targets completed additional monthly monitoring for reassessment. ‐ Conducted telephone consultations every 3 months for ongoing evaluation and health management.	‐ Home blood pressure monitoring. ‐ Personalized case management. ‐ Tailored support to effectively manage and improve their health outcomes.	‐ Before intervention. ‐ 3 months after intervention. ‐ 6 months after intervention. ‐ 9 months after intervention. −12 months after intervention.	‐ Systolic blood pressure. ‐ Diastolic blood pressure.	The adjusted odds ratio for ambulatory blood pressure monitoring BP target achievement was 1.48 (95% confidence interval 0.87–2.52, *p* value 0.15). At 12 months, the mean difference in BP changes between intervention and control groups was 1.6/1.1 for ambulatory blood pressure monitoring (*p* value 0.26 for systolic BP and 0.10 for diastolic BP), and 4.9/3.1 for home blood pressure monitoring (*p* value 0.04 for systolic BP and 0.01 for diastolic BP), favoring the intervention. Intervention group participants had hypotension (systolic BP < 110) more frequently (21% vs. 5%, *p* value 0.009), but no differences in orthostatic symptoms, syncope, nonmechanical falls, or emergency department visits.	‐ No insight details the difference in blood pressure. ‐ Should Integrate wearable technology for continuous remote monitoring of blood pressure. ‐ Lack of follow‐up phase to evaluate the sustainability of program. ‐ Lack of the discussion section by comparing the results of this study with other similar interventions.

Ongkulna et al. [38]/Thai	RCT: To investigate the effectiveness of the Gregory‐based self‐management education program (GBSEP) in enhancing health literacy, self‐efficacy, and self‐management behaviors among older adults with uncontrolled hypertension.	Level 1.c 10/13 High	Community/ ‐ Intervention group total 50 people, male 17 (34.0%), female 33 (64.0%), mean age 67.98 ± 6.30 ‐ Control group total 50 people, male 15 (30.0%), female 35 (70.0%), mean age 68.16 ± 5.08 ‐ Older adult 100%	‐ Conducted a six‐session program (2 h each) using group discussions, videos, and hands‐on activities to enhance learning. ‐ Session 1: Introduced consequences of uncontrolled hypertension and encouraged reflection on personal experiences and beliefs. ‐ Session 2: Provided education on hypertension, complications, treatments, and self‐management using multimedia tools. ‐ Sessions 3–6: Focused on practical skills—DASH diet planning, exercise for older adults, medication and emotion management, and relaxation techniques. Included interactive activities such as dietary planning with food models and group exercises to promote real‐life application.	‐ Consequences of uncontrolled hypertension. ‐ Complications ‐ Treatments ‐ Proper self‐management techniques. ‐ DASH diet and dietary planning. ‐ Exercises. ‐ Medication management. ‐ Emotion management and relaxation techniques.	‐ Before intervention. ‐ 3 weeks after intervention. ‐ 1 month after intervention finished. ‐ 3 months after intervention finished.	‐ Health literacy. ‐ Self‐efficacy. ‐ Self‐management behaviors.	Result of baseline, end the program, 1 month and 3 months after program finished of the intervention/control group. ‐ Health literacy 36.26 ± 8.46, 44.56 ± 6.70, 45.62 ± 6.03, 46.94 ± 5.31/36.16 ± 8.60, 36.36 ± 8.70, 36.58 ± 8.54, 36.76 ± 8.60 *p* value < 0.001 ‐ Self‐efficacy 70.84 ± 10.74, 83.66 ± 9.34, 86.24 ± 8.52, 87.88 ± 8.00/69.40 ± 10.66, 69.44 ± 10.54, 69.82 ± 10.53, 69.96 ± 10.52 *p* value < 0.001 ‐ Self‐management behaviors 80.94 ± 9.95, 99.66 ± 8.04, 102.34 ± 8.10, 104.44 ± 7.09/79.24 ± 9.74, 79.60 ± 10.38, 79.88 ± 10.26, 80.08 ± 10.22 *p* value < 0.001	‐ Ensure that the outcomes assessors were blind to the treatment assignments. ‐ Lack of detailed description of the routine patient education. ‐ Incorporate qualitative feedback from participants about their experiences with the program. ‐ Implement of technology, therefore mobile apps, or online platforms, to deliver the Gregory‐based education.

Kohn et al. [41]/United state of America	RCT: To evaluate the effects of a 12‐week randomized, single‐blind Tai Chi (TC) intervention on deficit frailty in community‐dwelling older adults with hypertension.	Level 1.c 9/13 Moderate	Community/ ‐ Health education group total 80 people, male 26 (32.0%), female 54 (68.0%), mean age 73.27 ± 7.7 ‐ Tai Chi group total 87 people, male 25 (28.0%), female 62 (72.0%), mean age 71.6 ± 7.5 ‐ Older adult 100%	‐ Conducted Tai Chi sessions twice weekly (60 min each) for 12 weeks, including warm‐up, 40 min of guided practice, and cool‐down totaling 24 h of supervised training. ‐ Led by a certified instructor using the “moving for better balance” curriculum to enhance posture, mindfulness, coordination, and mobility. ‐ The health education (HAP‐E) group met weekly for 120 min, covering multidimensional topics such as sleep, nutrition, cardiovascular health, diabetes prevention, mental health, and resilience. ‐ Each HAP‐E session included a lecture, group discussion, and problem‐solving activities to support knowledge sharing and practical application.	‐ Tai Chi Exercise Health education ‐ Nutrition. ‐ Cardiovascular. ‐ Diabetes prevention. ‐ Mental health ‐ Socialization and resilience	‐ Before intervention. ‐ 12 weeks after intervention.	‐ Frailty index. ‐ Systolic blood pressure. ‐ Diastolic blood pressure.	‐ Frailty index (FI) scores in the HAP‐E group, 29 participants (36%) were frail. In Tai Chi group, 27 participants (31%) were frail. OR 1.26 (0.63, 2.53) ‐ Systolic blood pressure HAP‐E group 135.7 ± 20.2 Tai Chi group 134.1 ± 16.3 d = 0.09 (−0.23, 0.41) ‐ Diastolic blood pressure HAP‐E group 69.3 ± 10.0 Tai Chi group 69.5 ± 9.6 d = −0.02 (−0.33, 0.29)	‐ No details about changes in each variable were provided, as the results were reported only briefly. ‐ Limited sample size, potentially affecting the generalize of the findings. ‐ Lack of a long‐term follow‐up to assess the sustainability. ‐ Lack of diversity in participant demographic. ‐ Inadequate description of detailed descriptions of the Tai Chi and health education protocols.

Sun et al. [42]/China	RCT: To develop a health behavioral digital intervention for hypertensive patients (HBDIHP) based on an intelligent health promotion system and WeChat following the behavior change wheel (BCW) theory and digital microintervention care (DMIC) model and assess its efficacy.	Level 1.c 12/13 High	Community/A total of 54 participants (30 women and 24 men; mean age 67.24 ± 4.19 were included in the final analysis: 23 in the experimental group and 31 in the control group. ‐ Older adult 100%	‐ Developed a behavior change wheel (BCW)–based digital intervention delivered via WeChat. ‐ Provided weekly educational videos on exercise, diet, medication, and blood pressure monitoring. ‐ Applied BCW strategies: persuasion through behavior identity reflection, incentivization with motivational imagery, and training via practice videos and self‐scoring. ‐ Included environmental structuring to encourage physical activity and enablement through goal setting, feedback, and habit tracking. ‐ Program phases: Week 1 preparation, Weeks 2–6 commitment and planning, and Weeks 7–12 reinforcement of behavioral habits.	‐ Exercise. ‐ DASH diet. ‐ Medication adherence. ‐ Blood pressure monitoring.	After intervention ‐ Week 1 ‐ Week 2 ‐ Week 3 ‐ Week 4 ‐ Week 5 ‐ Week 6 ‐ Week 7 ‐ Week 8 ‐ Week 9 ‐ Week 10 ‐ Week 11 ‐ Week 12	‐ Systolic blood pressure. ‐ Diastolic blood pressure. ‐ Medication adherence.	Result of baseline and 12 weeks after intervention of intervention/control group. ‐ Systolic blood pressure 135.43 ± 17.48, 125.74 ± 14.76 *p* value 0.002/136.94 ± 18.44, 133.10 ± 15.02 *p* value 0.10 ‐ Diastolic blood pressure 78.39 ± 8.81, 75.96 ± 6.38 *p* value 0.19/76.03 ± 9.20, 75.58 ± 6.94 *p* value 0.129 ‐ Medication adherence 6.77 ± 1.22, 7.65 ± 0.49/6.99 ± 1.02, 7.09 ± 1.00 *p* value 0.46	‐ The study uses WeChat that involves the two‐way communication, and the participants could gain more media including VDOs, health education infographics, or manuals

#### 7.4.2. Dietary Approach to Stop Hypertension (DASH Diet)

The DASH diet was specifically highlighted as a key dietary strategy across multiple interventions. The DASH diet was found in six articles that mentioned implementing a nutritional education support program that emphasized the DASH diet’s principles, focusing on increased intake of fruits, vegetables, whole grains, and low‐fat dairy products while reducing the consumption of saturated fats, cholesterol, and refined sugars. The program used experiential learning techniques such as group discussions and practical food preparation activities, enabling participants to adopt DASH diet habits in their daily lives [30–32, 38, 39, 42]. This approach resulted in significant reductions in both SBP and DBP. DASH diet education was also integrated into the program, utilizing multimedia instruments such as videos, PowerPoint presentations, and dietary planning exercises to enhance understanding and adherence. These structured educational sessions effectively promoted long‐term dietary changes that supported blood pressure control.

#### 7.4.3. Exercise Promotion

Exercise promotion was another central element of hypertension management. Exercise promotions were found in 11 articles focused on enhancing physical activity to improve cardiovascular health and lower blood pressure. To implement a nurse‐led hypertension management program that included physical activity training sessions tailored to older adults’ capabilities [33]. These sessions emphasized moderate aerobic exercises, focusing on advice for walking, stretching, and strength training to improve overall physical fitness. The program included group exercise sessions and individualized activity plans to encourage consistent physical activity [27, 28, 30, 32, 35, 38–42]. Aerobic exercise, resistance training, flexibility exercises, and mind‐body practices like Tai Chi were mentioned. Aerobic activities such as walking and structured physical training, while resistance training, enhance cardiovascular health and muscle strength. Flexibility and balance exercises, including stretching and Tai Chi, can improve mobility and stress reduction. Moderate‐intensity exercise such as brisk walking or Tai Chi, performed for 30–60 min per session, at least five days per week, is widely recommended. Resistance training is typically conducted twice weekly, with one to two sets of 10–15 repetitions, while flexibility and balance exercises are encouraged two to three times per week [41]. Despite its benefits, exercise adherence among older adults remains challenging due to mobility limitations and cognitive decline. Community‐based programs incorporating personalized coaching, home visits, and digital interventions, such as mobile health applications and telemonitoring, have been used to improve engagement [36]. Social support through group‐based exercise sessions has also proven effective in sustaining long‐term physical activity [42]. Given these findings, this study aims to review and summarize hypertension management interventions focusing on exercise programs, identifying optimal strategies to enhance adherence, and achieving sustainable blood pressure control in older populations. The intervention demonstrated significant reductions in SBP and DBP.

#### 7.4.4. Weight Control

Weight control was indirectly addressed through interventions promoting healthy dietary behaviors and increased physical activity. The primary focus was on improving dietary and physical activity, with body weight considered an outcome indicator rather than the main focus. Integrating DASH‐diet principles and regular exercise facilitated gradual weight loss and maintenance, which contributed to improved blood pressure outcomes. Although none of the studies focused solely on weight control, the holistic lifestyle interventions reduced the body mass index and waist circumference, indirectly supporting hypertension management. The structured approach incorporating dietary modifications, physical activity, and behavioral support has been found effective in achieving weight loss and sustaining long‐term cardiovascular benefits. Nutritional interventions focusing on balanced, low‐sodium diets, such as the DASH diet, have been widely recommended for weight control and hypertension management [31, 32, 38, 39, 42]. Cooking workshops and individualized dietary counseling have been implemented to enhance adherence and empower individuals with the knowledge and skills necessary to maintain a healthy diet [28]. Weight controls were reported in an article that mainly mentioned the DASH diet and exercise promotion. However, 11 articles mainly report on weigh control and self‐care to control body weight, which focus on the health behavioral support and lifestyle modification [27, 28, 31, 33, 34, 36–40, 42].

#### 7.4.5. Smoking Cessation

Smoking cessation was explicitly addressed in several interventions as a crucial lifestyle modification for hypertension management. Smoking cessations were found in seven articles that mentioned incorporating smoking cessation education within the long‐message service and phone‐based health‐coaching program, emphasizing the risks of smoking and its direct impact on hypertension. Participants received tailored educational messages detailing the cardiovascular risks associated with smoking along with practical strategies for quitting focusing on identifying smoking triggers, managing withdrawal symptoms, and adopting healthier coping mechanisms [27–30, 33, 35, 39]. The interventions increased the awareness of the health benefits of smoking cessation, including improved blood pressure control, and reduced cardiovascular complications. Additionally, participants were encouraged to set personal goals for quitting and received motivational support through regular follow‐up calls. Smoking cessation techniques were utilized in individual or group health education with consultation support, and this process was most reported to take 1–2 h. These structured educational and supportive interventions significantly improved smoking cessation rates, contributing to better hypertension management outcomes.

#### 7.4.6. Alcohol Cessation

Alcohol cessation was also addressed as a key lifestyle modification in managing hypertension. Alcohol cessations were found in six articles that mentioned that implementing educational interventions provided participants with knowledge about the adverse effects of excessive alcohol consumption on blood pressure and overall cardiovascular health. They also integrated alcohol cessation education into the long‐message service and phone‐based health‐coaching program, emphasizing the importance of moderating alcohol intake to maintain optimal blood pressure levels. Participants received evidence‐based guidelines on safe alcohol consumption limits. They were educated about the physiological effects of alcohol on the cardiovascular system, including its role in raising SBP and DBP. The intervention also focused on behavior modification techniques such as setting personal limits, tracking alcohol intake, and adopting alternative social activities that did not involve alcohol. Follow‐up sessions reinforced these educational messages, supported participants in overcoming challenges related to alcohol cessation, and provided personalized feedback on their progress [28–30, 33, 35, 39]. Alcohol cessation techniques were utilized in individual or group health education with consultation support. These processes were most reported to take time for 1–2 h. These strategies effectively reduced alcohol consumption among participants, contributing to better blood pressure control and overall cardiovascular health.

#### 7.4.7. Stress Management

Stress management has been a consistent and integral component of interventions aimed at improving hypertension control in older adults. These interventions were found in 10 articles that typically incorporated relaxation techniques mainly focusing on mindfulness meditation, deep breathing exercises, progressive muscle relaxation, and guided imagery, which were often delivered through structured programs lasting 12 weeks [27–33, 35, 39, 40]. Stress management strategies were frequently integrated into broader health education and self‐management initiatives, with participants receiving instruction during group sessions or one‐on‐one counseling, or via digital platforms such as WeChat. Therefore, mindfulness and relaxation techniques were taught in group discussions or skill‐building workshops, or through tailored messages in phone‐based coaching programs. Stress management emphasizes the importance of personalized support, with follow‐up mechanisms such as weekly or monthly telephone calls, home visits, or digital reminders to reinforce stress management practices and ensure adherence. Programs like the self‐care behavior‐promoting program [28] and the Gregory‐based self‐management education program [38] included practical activities such as role‐playing and logbook tracking to help participants integrate stress reduction techniques into their daily routines. Family involvement and multidisciplinary approaches were often employed to create a supportive environment for sustained stress management. Overall, these interventions highlight the critical role of stress reduction in hypertension control, with consistent evidence supporting relaxation techniques, education, and personalized follow‐up to improve blood pressure outcomes and overall well‐being in older adults.

#### 7.4.8. Medication Adherence

Medication adherence was found in 10 articles that mentioned implementing various strategies to enhance adherence. Educational programs, personalized coaching, and technological support were commonly used to address barriers to consistent medication use. Tailored interventions, such as customized long‐message services and phone‐based health coaching, provided reminders and education on medication adherence’s importance, significantly improving adherence rates. Self‐management education programs, often delivered through face‐to‐face and telephone‐based sessions, emphasize proper dosing and the risks of non‐adherence while also addressing health literacy barriers [27, 29, 30, 32–35, 37, 39, 42]. Nurse‐led programs incorporate regular monitoring and counseling to ensure consistent medication use, which is often supported by family involvement. Multidisciplinary approaches combined educational materials, electronic pill boxes with reminders, and monthly pill counts to track and reinforce adherence were also used. Patient‐centered communication programs focused on goal setting, action planning, and follow‐ups to reinforce medication‐taking behaviors, while self‐management interventions utilized home visits and group sessions to provide ongoing support and education. Digital health interventions delivered via WeChat offered educational content, reminders, and self‐monitoring tools to enhance adherence. Finally, telemonitoring and protocolized case management further supported adherence through home blood pressure monitoring and pharmacist‐led counseling.

### 7.5. Effects of Intervention Delivery Strategies on Blood Pressure Outcomes

Across the 16 included studies, a comparative pattern of 12 related studies was observed: Interventions delivered through combined multicomponent strategies produced the largest and most consistent reductions in blood pressure [27–33, 37, 39–42], whereas single‐component approaches showed smaller, less consistent, or nonsignificant effects. Blood pressure findings were examined across four delivery strategies within the intervention component framework including health education, consultation support, health monitoring, and combined approaches integrating multiple strategies (Tables 4 and 5) and interpreted alongside dose characteristics, including frequency, duration, session length, fidelity, and measurement time point (Table 6), as well as extracted SBP and DBP outcomes (Tables 7 and 8). Nevertheless, the comparative pattern indicated that effectiveness increased with greater integration of interventions, particularly when multiple delivery modalities were combined.

**TABLE 5:** Descriptive mapping of intervention components from the included papers.

Study	Nutrition/DASH	Exercise/weight control	Smoking cessation	Alcohol cessation	Stress management	Medication adherence	Health monitoring	Health‐education	Consultation	Combined strategies	SBP improved
Anantasaran [28]	✓	✓	✓	✓	✓			✓	✓	✓	Favors
Kim [27]	✓	✓	✓	✓	✓	✓		✓	✓	✓	Favors
Delavar et al. [29]						✓	✓	✓	✓		Favors
Kolcu and Ergun [30]	✓	✓	✓	✓	✓	✓	✓	✓	✓		Favors
Kitrungrote [31]	✓				✓		✓	✓			Not report
Woodham et al. [32]	✓	✓	✓	✓	✓	✓		✓		✓	Favors
Audthiya et al. [33]					✓			✓	✓		Not report
Putri et al. [35]	✓	✓	✓	✓	✓			✓	✓		Not report
Zhang et al. [34]	✓	✓	✓	✓	✓	✓	✓	✓	✓		Favors
Sukpattanasrikul et al. [39]		✓	✓	✓			✓	✓		✓	Favors
Wong et al. [36]								✓	✓	✓	Favors
Bumrungsuk [40]	✓	✓	✓	✓	✓			✓	✓		Favors
Lau et al. [37]							✓	✓	✓		Not report
Ongkulna et al. [38]	✓	✓	✓	✓	✓			✓			Not report
Kohn et al. [41]	✓	✓	✓	✓	✓			✓			Not report
Sun et al. [42]	✓	✓	✓	✓		✓	✓	✓			Favors

Abbreviation: SBP = Systolic blood pressure.

**TABLE 6:** Extraction of intervention dose characteristics across included papers.

Study	Arm (*n*)	Frequency	Duration (weeks)	Session length	Fidelity	Measurement timepoint	Primary endpoint
Anantasaran, 2016	Health‐promoting/self‐care behavior–promoting program (30) vs usual care (30)	Two group sessions during Weeks 1–2 + 1 home visit during Weeks 3–8 + telephone follow‐up two times	10	3 h/session (group sessions); home visit 45–60 min	Health belief model + social support; BP assessment, health education, self‐care skill training, home visit, telephone reinforcement	Baseline, 10 weeks	Self‐care behavior and blood pressure
Kim [27]	Health‐coaching (30) vs control (31)	One session/week	8	30 min/session	Nurse‐delivered; > 40‐h training; structured IMCHB coaching	Baseline, 8 weeks	Blood pressure reduction
Delavar et al. [29]	SME tailored to HL (56) vs control (58)	Two face‐to‐face/week (first 2 weeks) + 2 calls/week	6	30–45 min (face‐to‐face); 15 min (phone)	HLI‐based materials; teach‐back method; expert‐validated (HLI score 84%)	Baseline, 6 weeks	Medication adherence; SBP
Kolcu and Ergun [30]	Nurse‐led HT management program (37) vs control (37)	Weekly meetings during intervention; six education sessions + 4 brief motivational meetings	20	NR	Individual + group + institutional actions; BP/anthropometric repeated at motivational meetings; make‐up individual education for nongroup participants; medicine boxes; saltshaker removal; exercise scheduling; DASH implementation	Pretest, post‐test (4 weeks after completion); intervention	SBP, DBP, HT knowledge, quality of life, medication adherence
Kitrungrote, [31]	Nutritional educational support program (36)	NR	7	NR	Experiential learning (Kolb) + group process + DASH‐based program	Week 1, Week 8, Week 15	Nutritional behavior
Woodham et al. [32]	Multidisciplinary (100) compared to control (100)	NR	12	NR	Multidisciplinary lifestyle + adherence program	Baseline, 4 weeks, 12 weeks	SBP change over time
Audthiya et al. [33]	PCC program (30) vs control (30)	Weekly sessions + telephone follow‐up	12	30–90 min/session	Structured PCC model: communication training, role‐play, goal setting, counseling, follow‐up	Baseline, 12 weeks	Autonomy, self‐management behaviors
Putri et al. [35]	Self‐management (67) vs control (67)	Two sessions/week (home visits)	2	55–60 min/session	Structured 4‐session program: education, physical activity, diet, relaxation, adherence + caregiver involvement + workbook/module	Baseline, 2 weeks	Self‐care adherence, health status
Zhang et al. [34]	RAM‐based nursing (60) vs control (60)	Continuous during hospitalization + monthly follow‐up × 3	12	NR	Structured RAM framework (physiological, self‐concept, role, interdependence); individualized assessment + tailored intervention + family involvement + follow‐up	Baseline, 12 weeks	Self‐management, adherence, QoL, BP control
Sukpattanasrikul et al. [39]	Self‐management program (78) vs control (78)	Weekly sessions (weeks 1–4) + periodic follow‐up (weeks 5–16)	16	30–60 min/session	IFSMT‐based SMP; group sessions + caregiver involvement + telephone follow‐up + skill training	Baseline, 4, 8, 12, 16 weeks	SBP, DBP, self‐care, QoL
Wong et al. [36]	mHealth + I with nurse support (74) vs control (76)	Daily app input/review + 8 proactive nurse calls over 3 months + biweekly case conferences	12	NR	Nurse case management supported by health–social partnership team; daily review of app entries; standardized protocols; eight proactive calls	Baseline, 12 weeks	QoL (primary); SBP secondary
Bumrungsuk [40]	Self‐management training (44) vs control (47)	One session/week (small group) + home visits (2 follow‐ups)	13	30–60 min/session	Creer‐based self‐management: education + goal setting + self‐monitoring + group discussion + home visit reinforcement	Baseline, 4 weeks, 13 weeks	Self‐management behavior (primary); BP secondary
Lau et al. [37]	Telemonitoring + pharmacist case management (61) vs HBPM‐only (59)	HBPM daily (7‐day series every 3 months); care manager contacts every 3 months	48	Not fixed (telephone visit; self‐monitoring)	High: telemonitoring + pharmacist protocolized medication titration + adherence support	Baseline, 48 weeks	Proportion achieving SBP target
Ongkulna et al. [38]	Gregory‐based self‐management education (50) vs Routine education (50)	Two sessions/week	12	2 h/session	High: structured Gregory + transformative learning + group activities + booklet	Baseline, 4, 12 weeks	Health literacy, self‐efficacy, self‐management behaviors
Kohn et al. [41]	Tai Chi (87) vs health education (80)	2 ×/week Tai Chi vs 1 ×/week (HAP‐E)	12	60 min/session Tai Chi/120 min/session (HAP‐E)	Moderate–high: instructor‐led, structured curriculum, attendance tracked	Baseline, 12 weeks	Frailty index
Sun et al. [42]	Digital intervention (23) vs usual care (31)	Continuous (daily microinterventions via WeChat; weekly adherence tracking)	12	Not explicitly fixed (microintervention, event‐driven delivery)	High: BCW + DMIC framework, personalized algorithm, weekly monitoring, adherence tracking	Baseline, 12 weeks	Blood pressure + medication adherence

*Note: n* = amount of sample, vs = versus, HT = hypertension, HAP‐E = healthy aging practice center education, IFSMT = individual and family self‐management theory.

Abbreviations: BCW = Behavior change wheel, DBP = diastolic blood pressure, DMIC = digital microintervention care, HBPM = home blood pressure monitoring, HL = health literacy, HLI = health literacy index, IMCHB = interaction model of client health behavior, PCC = patient center communication, QoL = quality of life, RAM = Roy adaptation model, SBP = systolic blood pressure, SME = self‐management education, SMP = self‐management program.

**TABLE 7:** Systolic blood pressure extraction between intervention and control groups across included studies.

Study	Baseline mean ± SD	Postintervention mean ± SD	MD intervention group	MD control group	Between‐group difference MDIG − MDCG	Between‐group difference at post‐test	*p* value	Confidence interval
Anantasaran, [28]	154.50 ± 10.11 vs 153.78 ± 9.87	140.10 ± 11.57 vs 148.96 ± 10.23	−14.40	−4.82	−9.58	−8.86	0.001	NR
fKim [27]	141.16 ± 18.28 vs 140.03 ± 11.60	132.53 ± 8.92 vs 143.58 ± 9.49	−8.63	3.55	−12.18	−11.05	< 0.001	NR
Delavar et al. [29]	156.43 ± 12.38 vs 154.22 ± 13.73	142.59 ± 11.56 vs 148.45 ± 12.32	−13.84	−5.77	−8.07	−5.86	0.004	NR
Kolcu and Ergun [30]	129.18 ± 14.60 vs 119.18 ± 15.16	118.64 ± 10.04 vs 130.54 ± 15.08	−10.54	11.36	−21.90	−11.90	< 0.001	NR
Kitrungrote, [31]	NR vs NR	NR vs NR	−14.00	NR	NR	NR	< 0.001	NR
Woodham et al. [32]	154.51 ± 11.15 vs 156.05 ± 9.77	141.27 ± 13.59 vs 143.56 ± 14.59	−13.24	−12.49	−0.75	−2.29	< 0.001	NR
Audthiya et al. [33]	NR	NR	NR	NR	NR	NR	NR	NR
Putri et al. [35]	NR	NR	NR	NR	NR	NR	NR	NR
Zhang et al. [34]	NR	135.77 ± 7.56 vs 142.50 ± 11.06	NR	NR	NR	−6.73	0.001	NR
Sukpattanasrikul et al. [39]	150.23 ± 7.10 vs 150.88 ± 6.22	133.91 ± 13.30 vs 149.17 ± 9.17	−16.32	−1.71	−14.61	−15.26	< 0.001	−18.43 to −11.09
Wong et al. [36] (mHealth + I vs Control)	136.27 ± 2.09 vs 136.83 ± 2.73	132.95 ± 2.34 vs 137.81 ± 3.05	−3.32	0.98	−4.30	−4.86	0.030	−4.25 to −0.35
Bumrungsuk [40]	138.27 ± 18.57 vs 132.49 ± 15.93	130.45 ± 26.30 vs 124.47 ± 14.31	−7.82	−8.02	0.20	5.98	0.040	0.28 to 12.59
Lau et al. [37]	132.5 ± 16.6 vs 132.8 ± 12.8	NR	−5.00	−3.20	−1.80	NR	0.256	−4.30 to 1.10
Ongkulna et al. [38]	149.70 ± 6.83 vs 151.24 ± 6.18	NR	NR	NR	NR	NR	NR	NR
Kohn et al. [41]	134.1 ± 16.3 vs 135.7 ± 20.2	NR	NR	NR	NR	NR	NR	NR
Sun et al. [42]	135.43 ± 17.48 vs 136.94 ± 18.44	125.74 ± 14.76 vs 133.10 ± 15.02	−9.69	−3.84	−5.85	NR	0.05	NR

Abbreviations: MD = Mean difference, NR = not reported, SD = standard deviation.

**TABLE 8:** Diastolic blood pressure extraction between intervention and control group across included studies.

Study	Baseline mean ± SD	Postintervention mean ± SD	MD intervention group	MD Control group	Between‐group difference MDIG − MDCG	Between‐group difference at post‐test	*p* value	Confidence interval
Anantasaran, [28]	99.82 ± 11.79 vs 98.85 ± 11.78	89.75 ± 10.85 vs 95.33 ± 10.58	−10.07	−3.52	−6.55	−5.58	0.005	NR
Kim [27]	86.61 ± 5.63 vs 88.06 ± 6.44	82.29 ± 4.79 vs 87.88 ± 8.12	−4.32	−0.18	−4.14	−5.59	0.019	NR
Delavar et al. [29]	96.11 ± 6.85 vs 96.03 ± 7.06	88.52 ± 7.99 vs 92.15 ± 9.33	−7.59	−3.88	−3.71	−3.63	0.023	NR
Kolcu and Ergun [30]	79.72 ± 9.57 vs 75.13 ± 10.17	77.83 ± 5.34 vs 82.70 ± 7.69	−1.89	7.57	−9.46	−4.87	0.003	NR
Kitrungrote, [31]	NR vs NR	NR vs NR	−8.22	NR	NR	NR	< 0.001	NR
Woodham et al. [32]	90.47 ± 6.13 vs 91.09 ± 5.57	73.22 ± 8.97 vs 76.69 ± NR	−17.25	−14.40	−2.85	−3.47	0.001	NR
Audthiya et al. [33]	NR	NR	NR	NR	NR	NR	NR	NR
Putri et al. [35]	NR	NR	NR	NR	NR	NR	NR	NR
Zhang et al. [34]	NR	73.81 ± 6.50 vs 77.65 ± 8.81	NR	NR	NR	−3.84	0.017	NR
Sukpattanasrikul et al. [39]	81.91 ± 9.36 vs 82.96 ± 9.47	77.03 ± 9.21 vs 86.35 ± 9.44	−4.88	3.39	−8.27	−9.32	< 0.001	−12.00 to −4.68
Wong et al. [36]	71.02 ± 1.16 vs 71.72 ± 1.24	70.41 ± 1.58 vs 71.44 ± 1.56	−0.61	−0.28	−0.33	−1.03	0.680	−4.02 to 2.63
Bumrungsuk [40]	73.75 ± 11.47 vs 75.96 ± 9.63	78.91 ± 13.36 vs 77.81 ± 11.35	5.16	1.85	3.31	1.10	0.510	−3.77 to 6.77
Lau et al. [37]	71.0 ± 8.8 vs 69.4 ± 8.6	NR	−3.30	−1.4	−1.9	NR	0.101	−2.40 to 0.20
Ongkulna et al. [38]	84.12 ± 6.06 vs 84.68 ± 5.13	NR	NR	NR	NR	NR	NR	NR
Kohn et al. [41]	69.5 ± 9.6 vs 69.3 ± 10.0	NR	NR	NR	NR	NR	NR	NR
Sun et al. [42]	78.39 ± 8.81 vs 76.03 ± 9.20	75.58 ± 6.94 vs 75.96 ± 6.38	−2.81	−0.07	−2.74	NR	0.840	NR

Abbreviations: MD = Mean difference, NR = not reported, SD = standard deviation.

A gradient in effect was observed across intervention delivery strategies when examining between‐group differences in SBP and DBP (Tables 4, 7, and 8). Interventions employing combined multistrategy approaches demonstrated the largest and most consistent reductions, with SBP reductions ranging from approximately −5.85 to −21.90 mmHg and DBP reductions from −2.74 to −9.46 mmHg. Within this category, multiple interventions achieved SBP reductions exceeding −10 mmHg, with a maximum observed reduction of −21.90 mmHg, accompanied by DBP reductions up to −9.46 mmHg [30, 39, 42].

Consultation‐supported interventions demonstrated moderate reductions in SBP, typically ranging between −8.07 and −12.18 mmHg, and in DBP, between −3.71 and −4.14 mmHg, reflecting clinically meaningful but comparatively smaller effects than fully integrated approaches [27, 29].

In contrast, health monitoring–focused interventions, particularly those relying on digital or telemonitoring systems, produced smaller and less consistent effects, with SBP reductions generally within −1.80 to −5.85 mmHg and DBP changes ranging from −0.33 to −2.74 mmHg, including nonsignificant findings, indicating limited standalone impact when not integrated with broader behavioral or consultation components [36, 37, 42].

Health education–only approaches showed the least consistent pattern, with minimal between‐group differences, SBP approximately −0.75 mmHg or incomplete reporting, limiting interpretability and suggesting insufficient effectiveness without reinforcement or follow‐up mechanisms [32].

## 8. The Subgroups Analysis of Outcomes

### 8.1. Effects of Intervention Duration on Blood Pressure Outcomes

Interventions conducted over approximately 10–16 weeks demonstrated the most consistent reductions in blood pressure, with SBP reductions ranging from −9.58 to −21.90 mmHg and DBP reductions from −6.55 to −9.46 mmHg [28, 30, 39].

Short‐duration interventions less than 6 weeks produced smaller reductions, with SBP changes around −8.07 mmHg and DBP changes approximately −3.71 mmHg, suggesting partial but less stable effects [29]. Conversely, extended‐duration interventions 48 weeks demonstrated minimal incremental benefit, with SBP reductions around −1.80 mmHg and DBP reductions approximately −1.90 mmHg, indicating diminishing returns beyond moderate‐duration exposure [37].

### 8.2. Effects of Intensity and Frequency on Blood Pressure Outcomes

Interventions characterized by repeated and structured contact were more effective than those with limited or undefined interaction. Programs with regular engagement achieved SBP reductions ranging from −8.63 to −16.32 mmHg and DBP reductions ranging from −4.32 to −8.27 mmHg [27, 39].

Higher‐intensity interventions combining multiple contact modes (e.g., face‐to‐face with telephone or digital follow‐up) consistently achieved SBP reductions exceeding −10 mmHg. In contrast, lower‐frequency or poorly defined interventions demonstrated smaller or inconsistent effects, typically below −5 mmHg SBP reduction [29, 36].

### 8.3. Effects of Session Length on Blood Pressure Outcomes

No consistent dose–response relationship was observed for session length. Effective interventions ranged from 30‐min sessions, achieving SBP reductions of approximately −8.63 mmHg, to longer sessions (2–3 h) associated with reductions up to −14.40 mmHg [27, 28]. These findings indicate that session duration does not affect blood pressure reduction. Instead, reductions of more than 10 mmHg in SBP were more consistently associated with structured programs that incorporated reinforcement and follow‐up.

### 8.4. Effects of Fidelity and Reinforcement on Blood Pressure Outcomes

Interventions with high fidelity and structured reinforcement mechanisms demonstrated more consistent and larger reductions, with SBP changes typically ranging from −9.69 to −16.32 mmHg and DBP reductions from −4.88 to −8.27 mmHg [39, 42]. However, fidelity alone was insufficient to guarantee large effects. Some high‐fidelity monitoring‐based interventions produced only modest SBP reductions, approximately −3.32 to −5.85 mmHg, and minimal DBP change, indicating that fidelity enhances blood pressure reduction primarily when combined with sufficient duration and contact intensity [36, 42].

### 8.5. Effects of Measurement Time Point on Blood Pressure Outcomes

The most consistent and clinically interpretable outcomes were observed at final follow‐up time points around 12 weeks, where SBP reductions ranged from −4.30 to −14.61 mmHg and DBP reductions ranged from −0.33 to −8.27 mmHg [36, 39]. Earlier time points showed smaller, more variable changes, indicating that sustained exposure to the intervention is required before stable reductions in both SBP and DBP can be achieved.

### 8.6. Effect on Secondary Outcomes

Several studies reported effects on secondary outcomes, including medication adherence [27, 29, 30, 32–35, 37, 39, 42], health behaviors [31–33, 35, 38–40, 42], hypertension knowledge [29, 30, 33, 38], psychological outcomes [30, 34, 36, 41], and selected biomarkers [32]. Compared with blood pressure outcomes, findings were more heterogeneous due to the variations in measurement and reporting.

The majority of studies assessing medication adherence reported improvements, particularly in interventions incorporating self‐management education (*n* = 16), behavioral support (*n* = 11), and monitoring (*n* = 9). Programs combining education with ongoing support demonstrated more consistent effects than single‐intervention approaches, although measurement variability limited comparability [35, 37, 42].

Health behaviors, such as diet [30–32, 38, 39, 42] and physical activity [27, 28, 30, 32, 35, 38–42], and hypertension‐related knowledge also improved in multicomponent interventions, showing that knowledge acquisition may support behavioral change. Psychological outcomes showed generally positive trends in interventions that included psychosocial or stress management components [27–33, 35, 39, 40].

Biomarker outcomes were infrequently reported and inconsistently measured [32], with some studies indicating favorable trends. Therefore, secondary outcomes showed positive but variable effects, and substantial heterogeneity limits conclusions regarding their consistency and magnitude. These findings showed that multicomponent interventions may influence behavioral and psychosocial factors to control blood pressure [32].

## 9. Discussion

Our findings highlight that effective hypertension management programs often employ a community‐based delivery model, leveraging social support networks and local health resources, consistent with global best practices. In Thailand, such interventions frequently involve community centers and village health volunteers who provide health education, nutritional counseling, physical activity programs, lifestyle modifications, and ongoing monitoring [28, 43]. These programs emphasize culturally tailored educational strategies focused on improving hypertension knowledge, self‐care behaviors, and risk perception. Community‐centric approaches enhance intervention reach and acceptability, foster peer support, and often include home visits, telephone follow‐ups, and personalized care. These elements align with Thai health policies that promote holistic care and family involvement, proving especially effective in facilitating behavioral change among older adults.

Across the included randomized trials [27, 29, 30, 33, 34, 36–38, 41, 42], most hypertension management programs demonstrated improvements in SBP and DBP as the primary outcome, which favors the intervention at approximately 12 weeks [29, 32, 34, 36, 41, 42]. The magnitude of within‐intervention SBP reduction ranged from 3.32 to 13.84 mmHg, while DBP reductions ranged from 0.6 to 7.6 mmHg. These findings suggest that multicomponent hypertension management interventions can achieve clinically meaningful reductions in blood pressure among community‐dwelling older adults. Programs incorporating self‐management education, lifestyle modification, and consultation support—such as nurse‐led management or digital‐assisted monitoring—appear particularly effective. For example, health literacy–tailored self‐management education substantially reduced both SBP and DBP, while nurse‐led and digital interventions also demonstrated consistent systolic reductions of approximately 9–10 mmHg within 12 weeks. Taken together, these observed findings suggest that interventions combining behavioral education, monitoring, and support strategies may enhance hypertension control in older adults by improving adherence to lifestyle and treatment recommendations.

Studies from other countries, including South Korea [27], Iran [29], Turkey [30], Indonesia [35], China [34, 42], Hong Kong [36], Canada [37], and the United States [41], reveal variations in hypertension management strategies shaped by regional guidelines and healthcare systems. These nine international studies reflect a shift toward multidimensional, theory‐based, and technology‐assisted interventions for community‐dwelling older adults. Moving beyond traditional education, these approaches emphasize personalized, data‐driven self‐management supported by behavioral science, digital health tools, and interprofessional collaboration to enhance patient engagement and continuous monitoring. Across the 16 reviewed articles, key interventions focused on stress management, medication adherence, and lifestyle modifications, aligning closely with guidelines from the World Health Organization [7], American Heart Association [6], and Thai Hypertension Society [43]. However, notable areas of both convergence and divergence with these guidelines were identified, as discussed below.

The reviewed studies consistently emphasized the importance of medication adherence as a cornerstone of hypertension management [30, 35, 37]. Interventions, including phone‐based coaching, educational programs, electronic pill reminders, and telemonitoring, effectively improved adherence rates [27, 34, 36, 37, 41]. These findings align with the WHO guidelines, which point out the need for patient education and support systems to enhance adherence [7]. Similarly, the AHA and the THS recommend the use of technology, such as mobile health applications and telemonitoring, to support medication adherence. However, while the reviewed studies often focused on short‐term adherence of three to 6 months, the WHO, AHA, and THA guidelines emphasize the importance of long‐term adherence strategies, including regular follow‐ups and patient engagement over the years [6, 7, 43]. This suggestion reflected a need for future interventions to incorporate more extended follow‐up periods to assess sustained adherence.

Stress management was a recurring theme in the reviewed studies, with mindfulness meditation, deep breathing exercises, and progressive muscle relaxation integrated into hypertension management programs. These approaches are supported by the AHA, which recognizes stress as a significant contributor to hypertension and recommends stress reduction techniques as part of lifestyle modifications [6]. The THS made no recommendation about the role of stress management in hypertension control. In contrast, the AHA provides specific recommendations, such as practicing mindfulness for 20–30 min daily [6, 43]. However, the reviewed studies provided evidence for the effectiveness of stress management; they often lacked detailed protocols for implementation, such as frequency and duration of sessions.

Lifestyle modifications, including dietary changes, physical activity, and weight management, were central to many reviewed interventions. Programs such as the DASH diet and Tai Chi were particularly effective in reducing blood pressure [31, 32, 38, 42]. These findings are consistent with the WHO guidelines, which advocate lifestyle changes as a first‐line treatment for hypertension [7]. The AHA and THS also emphasize the importance of a low‐sodium diet, regular exercise, and weight control in hypertension management. However, while the reviewed studies often focused on individual components of lifestyle modification, the AHA and THS recommend a comprehensive approach that combines diet, exercise, and stress management [6, 43]. These interventions suggest that future interventions could benefit from adopting a more holistic approach to lifestyle modification.

The use of technology, such as mobile health applications, telemonitoring, and electronic pillboxes, was a common feature in the reviewed studies [27, 34, 36, 37]. These tools effectively improved medication adherence, self‐monitoring, and patient engagement. This result aligns with the WHO, AHA, and THS guidelines, which endorse technology for hypertension management [6, 7, 43]. However, while the reviewed studies demonstrated these technologies’ short‐term benefits, WHO emphasizes the need for cost‐effectiveness analyses and long‐term sustainability of such interventions; this highlights a gap in the current literature as few studies assessed the economic feasibility or long‐term impact of technological interventions [7].

Self‐management and health education were key components of many interventions, with programs focusing on hypertension knowledge, self‐efficacy, and behavioral change. These findings align with the WHO and AHA guidelines, highlighting the importance of patient education in improving self‐management skills [6, 7]. The THS also emphasizes the need for culturally appropriate educational materials and community‐based programs [43]. However, while the reviewed studies often used group sessions and home visits for education, the AHA and THS recommend using digital platforms and community health workers to reach a broader participant [6, 43]. This suggestion reflected an opportunity for future interventions to leverage digital tools and community networks for education and self‐management support.

The findings from the 16 reviewed articles demonstrate significant alignment with the hypertension management guidelines from the WHO, AHA, and THS, particularly in the areas of medication adherence, stress management, lifestyle modifications, and the utilization of technology [6, 7, 43]. However, there are notable gaps in the current literature, including the need for long‐term follow‐up, cost‐effectiveness analyses, and holistic approaches to lifestyle modification. Therefore, hypertension management programs can achieve greater effectiveness, sustainability, and alignment with established best practices. In addition, future interventions should address these gaps while incorporating the detailed protocols and comprehensive strategies recommended by global and national guidelines.

We also found distinct intervention strategies for community‐dwelling older adults, including health education, consultation support, health monitoring, and a combination of these three approaches. These strategies significantly improved blood pressure, knowledge, health behaviors, medication adherence, risk perception, and biomarkers [6, 7]. Notably, interventions that integrated health education, consultation support, and health monitoring were the most effective in reducing high blood pressure and enhancing health perception [20]. While multiple intervention strategies exist, selecting the most appropriate approach requires the consideration of the target population and community context. Based on the review result, not all interventions were effective for all outcomes. Hypertension management programs incorporating technology‐based interventions may yield more effective outcomes for adults [6, 7, 43]. However, traditional community‐centered approaches may be more suitable for older adults, especially those with limited technological literacy. Nevertheless, community‐dwelling older adults proficient in using digital interventions when combining technology‐based interventions with conventional community care models may offer an optimal strategy for improving hypertension management outcomes.

A hierarchical pattern of intervention effect was observed. Multicomponent interventions integrating education, behavioral support, monitoring, and reinforcement consistently demonstrated the greatest reductions in blood pressure, a finding that aligns with large‐scale randomized trials and community‐based programs across diverse settings, including South Asia, Argentina, and Singapore, where integrated strategies significantly improved hypertension control [44–48]. Similarly, digitally enabled multicomponent interventions and structured self‐management programs combining monitoring, feedback, and behavioral support have demonstrated superior effectiveness compared to usual care or single‐component approaches [49, 50]. In contrast, consultation or coaching interventions alone produced moderate but less consistent effects, whereas education‐only interventions lacked sufficient impact to sustain blood pressure reduction. This gradient indicated that targeting multiple behavioral and clinical pathways simultaneously is more effective than addressing isolated components of hypertension management.

One of the most significant methodological contributions of this review is the integration of study quality into the interpretation of intervention effect. The convergence of consistent blood pressure reductions across higher‐quality randomized controlled trials implementing multicomponent interventions strengthens the inference that these strategies are not only effective but also likely causally related to improved hypertension control. Large cluster‐randomized trials and rigorously designed community interventions consistently demonstrate reductions in blood pressure when multicomponent strategies are employed [44–48, 51]. In contrast, studies with lower methodological rigor or less integrated intervention designs tend to show more variable or attenuated effects. While this review does not employ meta‐analytic pooling, the alignment of intervention complexity, study quality, and outcome consistency provides a robust causal argument supporting the superiority of multicomponent intervention designs.

Finally, the overall pattern of findings suggests that the effectiveness of hypertension management programs in community‐dwelling older adults is determined more by the integration of interventions than by any single component alone. Multicomponent interventions were more effective because they addressed multiple pathways simultaneously, including knowledge, adherence, self‐regulation, and ongoing behavioral reinforcement. In contrast, single‐component approaches appeared to exert a narrower and less sustained effect. This comparative gradient strengthens the interpretation that integrated, reinforced, and multistrategy interventions are more suitable for achieving meaningful blood pressure improvement in older adults than isolated or lower‐intensity approaches.

## 10. Limitations

This systematic review synthesizes international recommendations on hypertension management strategies for community‐dwelling older adults. However, limited guidance exists regarding the optimal format, design, and delivery of media or technology tailored to this population. In addition, substantial heterogeneity across included studies in intervention components, delivery strategies, duration, intensity, outcome measures, and follow‐up periods limited direct comparability and precluded meta‐analysis. Therefore, the relative effectiveness of interventions could not be determined through pooled estimates, and the findings should be interpreted as comparative patterns of effectiveness rather than precise measures of superiority.

## 11. Conclusions and Recommendations

This review of 16 studies demonstrates that the effects of hypertension management in community‐dwelling older adults are driven primarily by intervention integration rather than isolated components. Multicomponent interventions that combine health education, consultation support, and monitoring consistently produced the most reductions in blood pressure, reflecting their ability to target knowledge, adherence, and self‐regulation simultaneously. These findings indicate that targeting multiple behavioral mechanisms of knowledge, adherence, and self‐regulation is essential for sustained improvement. Clinically, interventions should prioritize integrated, reinforced, and context‐adapted models, with selective use of digital tools based on patient capacity. Future research should focus on component‐level effectiveness, intervention dose, innovation used, standardized blood pressure reporting, and long‐term outcomes. In summary, integration and continuity are the key determinants of effective hypertension management in older adults.

## Author Contributions

All authors contributed to the development of the study protocol, screening, data extraction, data analysis, data synthesis, reporting of results, discussion, and the drafting or revision of the manuscript. The specific contributions of each author are as follows:

Khanisorn Ransinyo: phenomenon exploration; formulation of the review aim and research questions; protocol development; literature searching; screening; consensus; data extraction; data analysis; data synthesis; result reporting; discussion; drafting and revising the manuscript; and submission.

Samoraphop Banharak: phenomenon exploration; formulation of the review aim and research questions; protocol development; literature searching; consensus; data extraction; data analysis; data synthesis; result reporting; discussion; drafting and revising the manuscript; and submission.

Chakkarin Sommana: literature searching; screening; consensus; data extraction; data analysis; data synthesis; result reporting; discussion; drafting and revising the manuscript; and submission.

## Funding

This systematic review was financially supported by the Fundamental Fund of Khon Kaen University through funding from National Science, Research, and Innovation Fund (NSRF) (Grant No.: NSRF68‐002). We appreciate this research funding institute for making the research possible.

## Disclosure

All authors have agreed on the journal to which the article will be submitted, provided final approval of the version to be published, and agreed to be accountable for all aspects of the work.

## Conflicts of Interest

The authors declare no conflicts of interest.

## Data Availability

The datasets generated and/or analyzed during the current study are not publicly available due to prohibited laws (and/or rules, regulations, and contracts). However, they are available from the corresponding author upon reasonable request.
